# Dynamic Catalysis Guided by Nucleic Acid Networks and DNA Nanostructures

**DOI:** 10.1021/acs.bioconjchem.2c00233

**Published:** 2022-08-16

**Authors:** Yu Ouyang, Pu Zhang, Itamar Willner

**Affiliations:** The Institute of Chemistry, The Hebrew University of Jerusalem, Jerusalem 91904, Israel

## Abstract

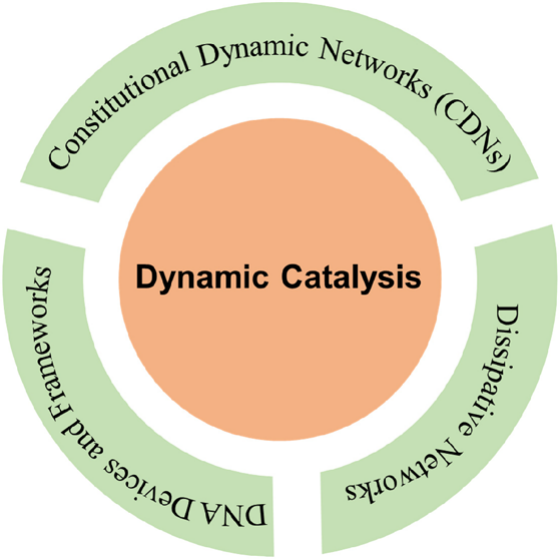

Nucleic acid networks conjugated to native enzymes and supramolecular DNA nanostructures modified with enzymes or DNAzymes act as functional reaction modules for guiding dynamic catalytic transformations. These systems are exemplified with the assembly of constitutional dynamic networks (CDNs) composed of nucleic acid-functionalized enzymes, as constituents, undergoing triggered structural reconfiguration, leading to dynamically switched biocatalytic cascades. By coupling two nucleic acid/enzyme networks, the intercommunicated feedback-driven dynamic biocatalytic operation of the system is demonstrated. In addition, the tailoring of a nucleic acid/enzyme reaction network driving a dissipative, transient, biocatalytic cascade is introduced as a model system for out-of-equilibrium dynamically modulated biocatalytic transformation in nature. Also, supramolecular nucleic acid machines or DNA nanostructures, modified with DNAzyme or enzyme constituents, act as functional reaction modules driving temporal dynamic catalysis. The design of dynamic supramolecular machines is exemplified with the introduction of an interlocked two-ring catenane device that is dynamically reversibly switched between two states operating two different DNAzymes, and with the tailoring of a DNA-tweezers device functionalized with enzyme/DNAzyme constituents that guides the dynamic ON/OFF operation of a biocatalytic cascade by opening and closing the molecular device. In addition, DNA origami nanostructures provide functional scaffolds for the programmed positioning of enzymes or DNAzyme for the switchable operation of catalytic transformations. This is introduced by the tailored functionalization of the edges of origami tiles with nucleic acids guiding the switchable formation of DNAzyme catalysts through the dimerization/separation of the tiles. In addition, the programmed deposition of two-enzyme/cofactor constituents on the origami raft allowed the dynamic photochemical activation of the cofactor-mediated biocatalytic cascade on the spatially biocatalytic assembly on the scaffold. Furthermore, photoinduced “mechanical” switchable and reversible unlocking and closing of nanoholes in the origami frameworks allow the “ON” and “OFF” operation of DNAzyme units in the nanoholes, confined environments. The future challenges and potential applications of dynamic nucleic acid/enzyme and DNAzyme conjugates are discussed in the conclusion paragraph.

## 1 Introduction

The information encoded in the base sequence of DNA includes substantial structural and functional information. This includes instructive structural reversible reconfiguration properties, such as dictated strand displacement of duplex structures,^1,2^ the reconfiguration of guanosine-rich strands, in the presence of K^+^-ions, into G-quadruplexes and their separation by crown-ethers,^3^ the pH-induced formation and separation of i-motif cytosine-rich sequences,^4^ the formation and dissociation of triplex nucleic acid structures,^5^ and the light-stimulated stabilization and dissociation of nucleic acid duplexes, by photoisomerizable intercalators, such as *trans*/*cis* azobenzene.^6,7^ Functional information dictated by the base sequence of nucleic acids includes sequence-specific recognition properties of low-molecular-weight substrates or macromolecules (aptamers)^8,9^ or sequence-guided catalytic functions of the nucleic acids, e.g., hemin/G-quadruplex DNAzymes^10,11^ or cofactor-dependent DNAzymes.^12,13^ The reversible reconfiguration of nucleic acids by auxiliary triggers was applied to develop diverse DNA switches^14^ and DNA machines,^15−17^ such as tweezers,^18,19^ walkers,^20−22^ interlocked catenated rings,^23,24^ and more.^25,26^ The recognition and catalytic functions of nucleic acids were broadly applied for the development of sensing platforms,^27−29^ amplifying agents for electrochemical or optical sensors,^30−32^ engineering of catalytic and photocatalytic supramolecular assemblies,^33−36^ and their therapeutic applications, including targeting of cells^37−39^ or inhibition of proteins,^40^ and more. In addition, the coded base-sequence of nucleic acids was extensively used to develop programmed linear structures and to assemble 2D^41,42^ and 3D nanostructures, such as DNA origami^41,43^ or DNA tetrahedra.^44^ Nucleic acid–protein conjugates or DNA–aptamer conjugates were engineered in spatially proximate positions on the DNA scaffolds to emulate biocatalytic cascades in confined native environments.^45^ Indeed, efficient bicatalytic cascades, as compared to an analog biocatalytically coupled reaction in nonorganized, random mixture of enzymes, were demonstrated.^46,47^

Furthermore, intracellular dynamic interactions between DNA, RNA, and proteins form complex biological networks triggered by environmental physical or chemical stimuli and operate under thermodynamic control or under transient, out-of-equilibrium, dissipative conditions. The intercommunication between these dynamic networks leads to programmed reaction patterns involving adaption,^48,49^ amplification,^50^ feedback^51^ or oscillatory^52^ mechanisms, signal propagation,^53^ switching,^54,55^ temporal and multistable behaviors,^56^ and clustering^48^ of networks into gated or cascaded assemblies. Diverse complex bioprocesses are driven by these networks, including cell differentiation,^48^ regulation of gene transcription,^57^ morphogenesis,^58^ and eventually to genome instability and stress-induced oncogene replication and cancer.^59−61^ In vitro design of chemical circuits mimicking natural dynamic networks attracts broad interest within the general topic of Systems Chemistry.^62−64^ The structural and functional properties of nucleic acids have been utilized to assemble dynamic DNA networks and reaction moduli triggered by different auxiliary triggers. These include the supramolecular engineering of constitutional dynamic DNA networks^65^ that demonstrated reversible adaptive,^66,67^ hierarchically adaptive,^68^ feedback-driven,^69^ and intercommunicating functionalities^70^ in the presence of auxiliary triggers. Also, transient, out-of-equilibrium, dissipative signal-triggered DNA-based frameworks were designed, including transcriptional oscillators^71^ or bistable regulatory networks^72^ and DNA networks coupled to biocatalysts, e.g., polymerase/endonuclease/nickase leading to oscillatory behavior.^73−75^ Also, fuel-triggered transient enzyme-coupled (nickase) networks,^76^ fuel-triggered transient DNAzyme systems,^77^ and light- or aptamer-guided transient ligation cycles^78,79^ were accomplished. Different applications of dynamically reconfigured DNA assemblies were introduced including the use of constitutional dynamic networks for controlling hydrogel stiffness for controlled drug release^80^ and for the dynamic network-guided release of drugs from nanocarriers.^81^ Transient DNA networks were applied as functional frameworks for the temporal uptake and release of loads,^82^ the photoacid-driven dissipative polymerization/depolymerization of DNA fibers,^83^ and the temporal aggregation/deaggregation of Au nanoparticles or CdSe/ZnS semiconductor quantum dots and the control over their optical properties.^84^

To emulate functions of native systems, it is important, however, to couple the trigged dynamic reconfiguration of DNA networks to dynamic biocatalytic processes. This can be accomplished by the conjugation of enzymes to dynamically triggered networks or by tethering biocatalysts to a dynamically switchable DNA framework. This Review discusses switchable catalytic properties and the control over biocatalytic cascades, by means of dynamic DNA networks conjugated to enzymes or by enzymes/DNAzymes conjugated to switchable DNA nanostructures.

### 2.1 Controlling Biocatalytic Cascades by Nucleic Acid-Based Constitutional Dynamic Networks (CDNs)

**Figure 1:**
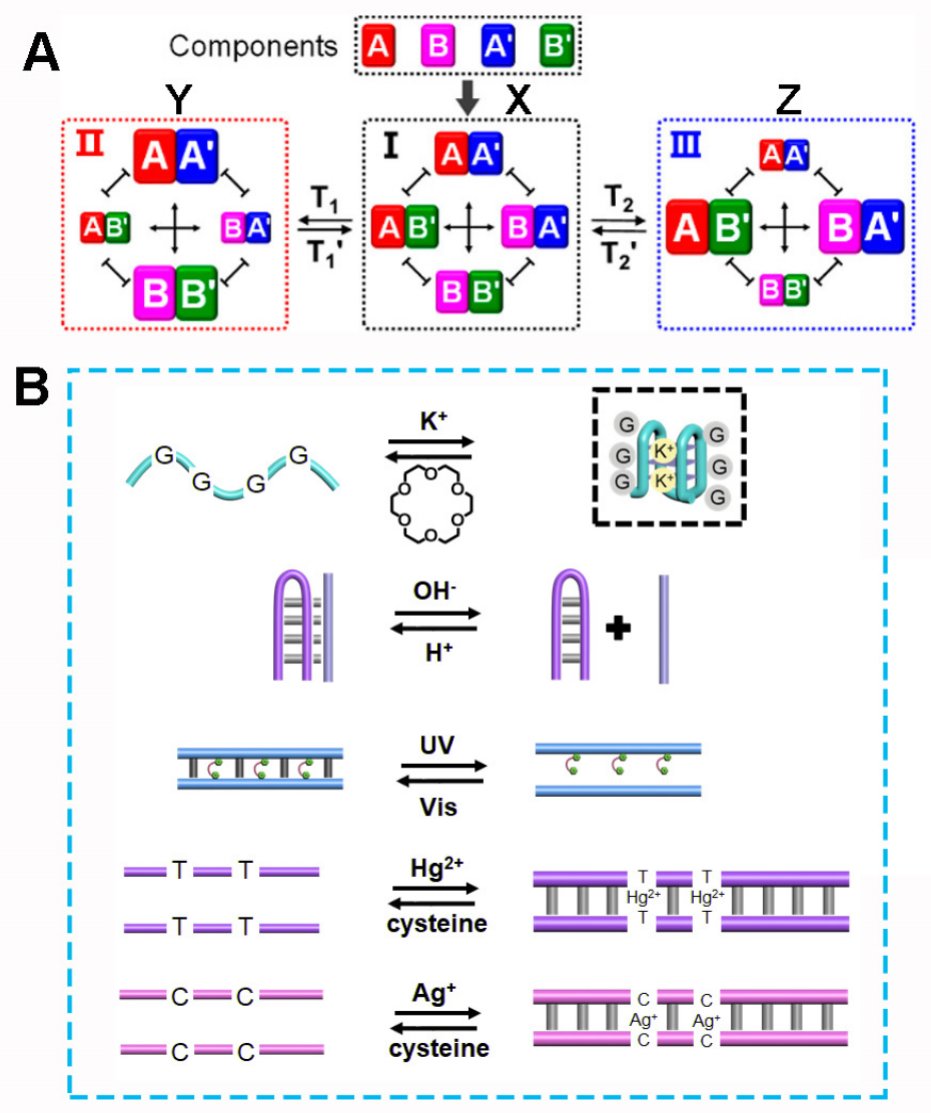
(A) Schematic assembly of a constitutional dynamic network (CDN) composed of four equilibrated constituents, CDN X, being dynamically reconfigured by auxiliary triggers. The constituents are composed of four components A, A′, B, B′, yielding the equilibrated constituents AA′, BB′, AB′, and BA′. The T_1_-triggered stabilization of constituent AA′ in CDN X leads to the dynamic reconfiguration of CDN X into the re-equilibrated CDN Y where AA′ is enriched at the expense of AB′ and BA′, resulting the concomitant enrichment of BB′ in the equilibrated mixture. Subjecting CDN X to trigger T_2_ stabilizes the constituent AB′ and as a result leads to the dynamic reconfiguration of CDN X to Z, where constituents AB′ and BA′ are enriched and constituents AA′ and BB′ are downregulated in their content in the resulting and BB′ re-equilibrated network. Adapted from ref (65) with permission. Copyright 2020, American Chemical Society. (B) Examples of reversible triggers that guide the programmed dynamic reconfiguration of CDNs.

Figure 1A depicts schematically the concept of a 2 × 2 constitutional dynamic network (CDN) composed of four constituents, AA′, BB′, AB′, and BA′. The four constituents exist in an equilibrated state X, where the contents of the constituents are controlled by their relative stabilities.^85^ Subjecting CDN X to an auxiliary trigger T_1_ that stabilizes, for example, constituent AA′ reconfigures CDN X into CDN Y where constituent AA′ is upregulated (increased in its equilibrated content) at the expense of constituents AB′ and BA′ that are downregulated and the concomitant increase in the content of BB′ (as a result of the recombination of B and B′ being separated upon supplying A and A′ to enrich AA′). Applying the counter trigger T_1_′ that destabilizes the constituent AA′ associated with CDN Y by displacement of T_1_ regenerates the equilibrated CDN X. Similarly, applying the trigger T_2_ that stabilizes constituent AB′ of CDN X reconfigures CDN X into CDN Z where AB′ is upregulated; the constituents AA′ and BB′, sharing components with AB′, are downregulated, and concomitantly, the constituent BA′ is upregulated. In addition, subjecting CDN Z to the trigger T_2_′, that destabilizes AB′, results in the recovery of the regenerated CDN X.

The information encoded in nucleic acids provides a versatile means to construct constituents of pre-engineered stabilities as scaffolds comprising the CDN. In addition, the nucleic acid constituents include in their scaffolds triggerable units that allow the reversible stabilization/destabilization of their structures (Figure 1B). These units might include a K^+^-ion G-quadruplex/crown ether switchable unit,^3^ a triplex stabilizing stand being displaced by a counter strand,^5^ a photoismerizable *trans*/*cis* azobenzene duplex intercalator stabilizing/destabilizing unit,^6,7^ or metal-ion bridged stabilized duplex, e.g., T-Hg^2+^-T or C-Ag^+^-C, being separated by a ligand such as cysteine.^86−88^ In addition, the constituent should be coupled to a functional element that quantitatively transduces content of the constituent upon the dynamic reconfiguration of the network. For example, by the integration of a catalytic nucleic acid into the structures of the constituents, e.g., Mg^2+^-ion-dependent DNAzymes, the rates of the DNAzyme-catalyzed cleavage of fluorophore-quencher modified substrates, and the use of appropriate calibration curves, provide a means to quantitatively report the contents of the constituents in the dynamic systems. Indeed, substantial progress was recently accomplished in designing and operating CDNs.^65^ Different triggers such as G-quadruplex formation and dissociation by K^+^-ions/crown ether,^67^ the formation and displacement of triplex nucleic acids,^89^ and the photoisomerization of azobenzene-modified intercalating strands^90^ were applied to reconfigure CDNs. Adaptive and hierarchically adaptive functions of dynamic networks,^68^ intercommunication^91^ and feedback-driven networks,^69^ and engineering of higher-order networks, such as three-dimensional networks,^92^ were demonstrated. Different applications of dynamic networks were introduced including the use of CDNs as functional units to stimulate dictated drug release^81^ or guided stiffness properties of hydrogel matrices and their use for switchable drug release and self-healing materials.^80^

Here we exemplify the conjugation of biocatalytic units to CDNs and the application of the hybrid matrices to stimulate dynamically controlled biocatalytic cascades and intercommunicated biocatalytic cascades. The operation of biocatalytic cascades in confined microenvironments of spatially proximate positions has been a subject of extensive experimental and theoretical research as a means to emulate biocatalytic processes in confined cellular media.^93,94^ The steric proximity between communicating catalysts where the product of one biocatalyst acts as the substrate of the neighboring biocatalyst proved to be an effective means to enhance biocatalytic cascades. Indeed, proximity effects induced by intercommunicated enzyme-loaded microdroplets,^95^ spatial positioning of biocatalysts on DNA scaffolds^96^ or origami tiles,^97^ or tethering enzymes on peptide or receptor frameworks^98,99^ proved a versatile means to spatially control biocatalytic cascades. In addition, the covalent tethering of biocatalysts on dynamically triggered DNA frameworks, e.g., tweezers, enabled the dynamic spatial control over the effectiveness of biocatalytic cascades.^100^ The covalent tethering of biocatalysts to constituents of CDNs allows not only the spatial control of proximity of intercommunicating biocatalysts but also the dynamic control over the content of the intercommunicating biocatalysts by means of the trigger reconfiguration of the DNA network.

**Figure 2:**
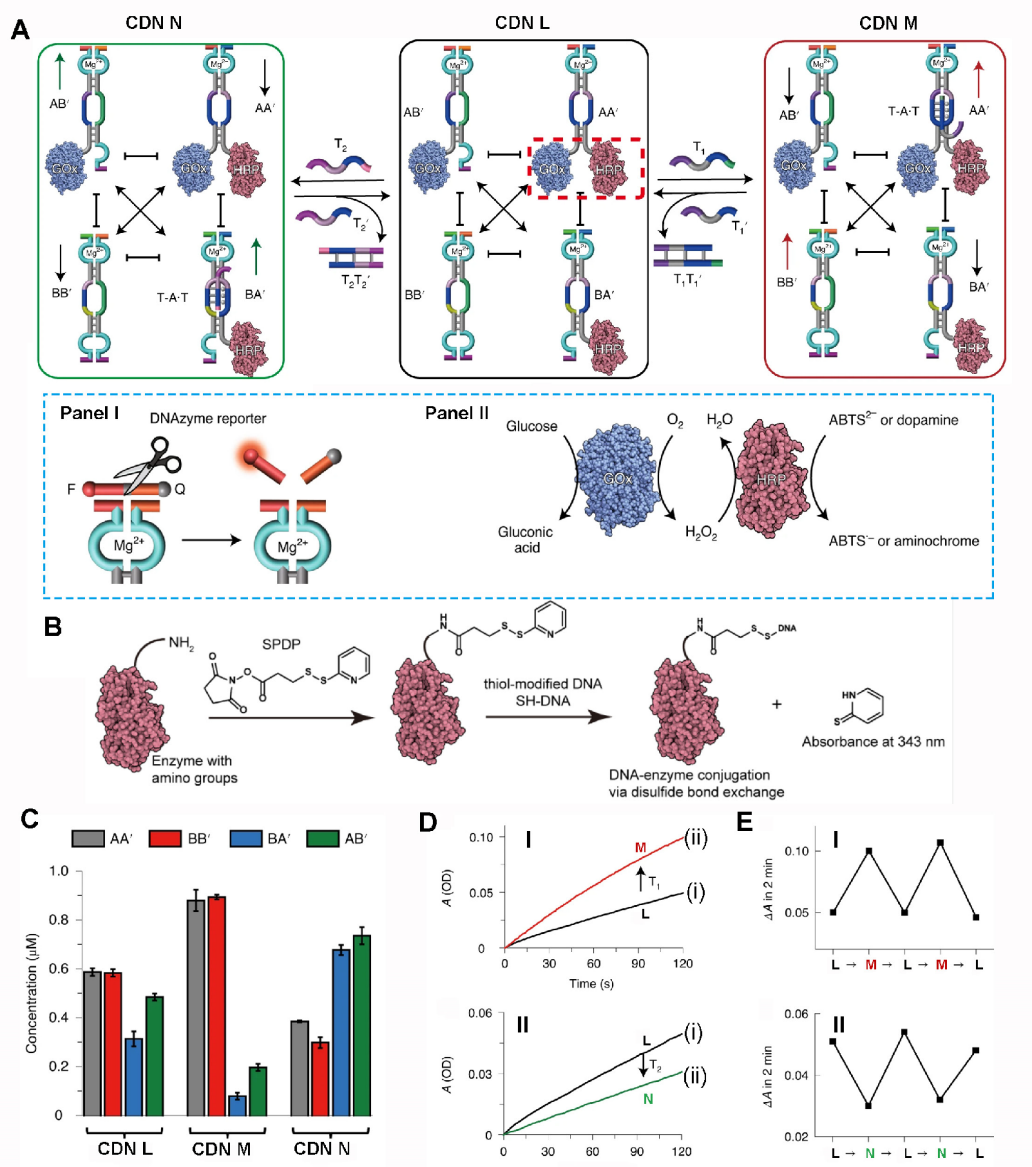
(A) Schematic nucleic acid-triggered operation of constitutional dynamic networks, CDNs, controlling the biocatalytic cascade consisting of GOx/HRP (see inset panel I). (B) Synthesis of enzymes functionalized with single nucleic acid tether A-GOx and A′-HRP. (C) Contents of the equilibrated constituents in CDN “L”, CDN “M”, and CDN “N”, evaluated by the activities of the Mg^2+^-ion-dependent DNAzymes associated with the constituents. (D) Time-dependent absorbance changes of ABTS^•-^, reflecting the efficacy of the GOx/HRP biocatalytic cascade associated with CDN “L”, panel I, curve (i), and T_1_-triggered reconfigured CDN “M”, panel I, curve (ii); Time-dependent absorbance changes of ABTS^•-^ reflecting the efficacy of the GOx/HRP biocatalytic cascade associated with CDN “L”, panel II, curve (i), and T_2_-triggered reconfigured CDN “N”, panel II, curve (ii). (E) Panel I-Cyclic operation of the GOx/HRP biocatalytic cascade upon the reversible switching, between CDN “L” and CDN “M” using the triggers T_1_ and T_1_′, and panel II-Cyclic operation of the GOx/HRP biocatalytic cascade upon the reversible switching, between CDN “L” and CDN “N” using the triggers T_2_ and T_2_′. Figure adapted from ref (101) with permission. Copyright 2020 Nature Publishing Group.

Figure 2A depicts the assembly of a DNA-based constitutional dynamic network, CDN L, that guides the dynamic spatial control over the intercommunicated cascade between glucose oxidase, GOx, and horseradish peroxidase, HRP.^101^ GOx is functionalized by nucleic acid component A, whereas the HRP is modified with the component A′. As a result, CDN includes four constituents, where each constituent includes a triggerable single-strand biloop domain, and each of the constituent is conjugated to a different Mg^2+^-ion-dependent DNAzyme reporter units (different arms corresponding to different fluorophore/quencher-modified substrates), acting as transducers for probing the contents of the constituents (Figure 2A, panel I). That is, the quantitative evaluation of the composition of the constituents in the equilibrated CDN is achieved by probing the catalytic activity of the different Mg^2+^-ion-dependent DNAzyme (catalytic rates) and using appropriate calibration curves relating the catalytic rates of the respective DNAzymes and variable concentration of the intact, respective DNAzymes. The constituent AA′ is thus functionalized with the spatially proximate GOx/HRP bicatalytic cascade where GOx catalyzes the aerobic oxidation of glucose to gluconic acid and H_2_O_2_, and the resulting H_2_O_2_ acts as a substrate for the intimately contacted HRP that catalyzes the oxidation of 2,2′-azino-bis(3-ethylbenzothiazoline-6-sulfonic acid) (ABTS^2–^) by H_2_O_2_ to form the colored product ABTS^•-^. The colored product ABTS^•-^ allows the spectroscopic probing of the GOx/HRP cascade (Figure 2A, panel II). The chemistry involved in the modification of the enzymes with a single nucleic acid is depicted in Figure 2B.

Treatment of CDN L with the triggering strand T_1_ results in the formation of a T-A•T triplex within constituent AA′, resulting in the stabilization of constituent AA′ and the reconfiguration of CDN L into CDN M, where AA′ and BB′ are upregulated and AB′ and BA′ are downregulated. The content of the spatially active GOx/HRP constituent, activating the biocatalytic cascade increases in the dynamically equilibrated CDN M. Subjecting CDN M to the counter strand T_1_′ separates the triplex unit, resulting in the reconfiguration of CDN M into CDN L. Similarly, subjecting CDN L to the triggering strand T_2_ that stabilizes a triplex domain in constituent BA′ reconfigures CDN L into CDN N where the constituent BA′ and AB′ are upregulated and the constituents AA′ and BB′ are downregulated, resulting in the CDN-guided decrease in the content of the biocatalytically active GOx/HRP cascade. In addition, triggering CDN N with the counter trigger T_2_′ restores the parent equilibrated CDN L. Figure 2C depicts the contents (concentrations) of the constituents in CDN L, the T_1_-triggered CDN M, and the T_2_-triggered, reconfigured, CDN N, reported by the activities of the Mg^2+^-ion-dependent DNAzymes coupled to the respective constituents. The contents of constituents AA′ and BB′ in CDN M increase by 49% and 53% and of AB′ and BA′ decrease by 58% and 76% as compared to the contents of their constituents in CDN L. Similarly, the reconfiguration of CDN L into CDN N is accompanied by the decrease of the contents of AA′ and BB′ by 35% and 48% and the increase in the concentrations of AB′ and BA′ by 54% and 100%, respectively. The triggered control over the concentrations of constituent AA′ in the three network controls the effectiveness of the GOx/HRP bicatalytic cascade (Figure 2D). The T_1_-triggered transition of CDN L to CDN M is accompanied by a 2.1-fold enhancement of the biocatalytic cascade, panel I, whereas the T_2_-triggered transformation of CDN L to CDN N results in a 0.6-fold inhibition of the biocatalytic cascade, panel II. By the reversible transition of CDN L to CDN M and CDN N by triggering T_1_/T_1_′ and T_2_/T_2_′, the activities of the respective biocatalytic cascade are cycled, and these follow the CDN-guided control over the concentrations of the enzymes (GOx and HRP) participating in the spatially intimate configuration that activates the bicatalytic cascades (Figure 2E, panel I and panel II). In analogy, a constitutional dynamic network driving the alcohol dehydrogenase, ADH, catalyzed the reduction of NAD^+^ to NADH, and the subsequent NADH mediated reduction of pyruvic acid to lactic acid, in the presence of lactate dehydrogenase, LDH, was achieved.^101^ The triggered reconfiguration of the CDN between upregulated and downregulated equilibrated configurations of the biocatalytic constituents was exploited, demonstrating the versatility of CDNs to control biocatalytic cascades.

**Figure 3:**
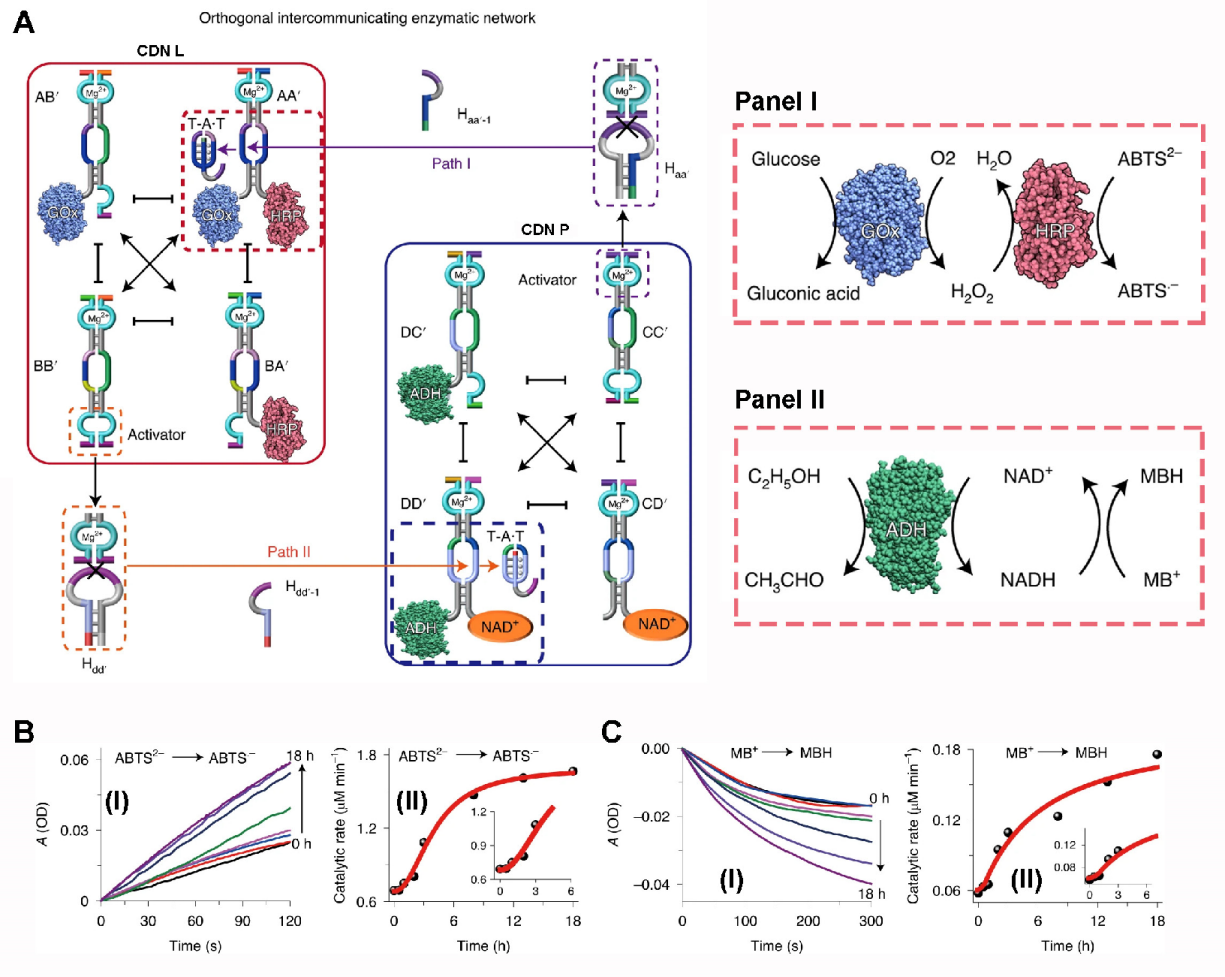
Orthogonal coupled operation of two constitutional dynamic networks that guide the dynamic cascaded biocatalytic reactions consisting of GOx/HRP and ADH/NAD^+^. The two CDNs “L” and “P” are subjected to two hairpins H_aa_′ and H_dd_′ that fuel the coupled operation of the networks. Cleavage of H_aa_′ by constituent CC′ associated with CDN “P” yields fragment H_aa-1_ that stabilizes constituent AA′ in CDN L resulting in the temporal dynamic enrichment of AA′ and the concomitant enrichment of constituent BB′. Simultaneously, cleavage of hairpin H_dd_′ by constituent BB′ associated with CDN L yields strand H_dd-1_′ that stabilized constituent DD′ of CDN P and the concomitant dynamic enrichment of constituent CC′. The operation of the two networks leads to the dynamic temporal enrichment of AA′ associated with CDN L and to the accompanying temporal feedback-driven enhancement of the GOx/HRP cascade (Figure 3A, panel I), and the concomitant temporal feedback-driven enrichment of constituent DD′ of CDN P accompanied by the temporal enhancement of the ADH/NAD^+^ cascade followed by the reduction of Methylene Blue, MB^+^, to reduced Methylene Blue, MBH (Figure 4, panel II). (B) Panel I: Time-dependent rates of ABTS^•-^ formation by temporal samples of the reaction mixture generated by the reaction mixture. Panel II: Catalytic temporal rates of ABTS^•-^ formation by the dynamically feedback-driven coupled CDNs system. (C) Panel I: Time-dependent rates of MBH formation by temporal samples of the reaction mixture generated by the ADH/NAD^+^ cascade. Panel II: Catalytic temporal rates of MBH formation by the dynamically feedback-driven coupled CDNs system. Figure adapted from ref (101) with permission. Copyright 2020 Nature Publishing Group.

Most importantly, however, the two CDNs-guided biocatalytic cascades comprising GOx/HRP and ADH/NAD^+^ were coupled to yield the CDNs-driven dynamic feedback operation of the two cascades (Figure 3A). Two hairpins, H_aa_′ and H_dd_′, were introduced into the mixture of CDN P and CDN L. Constituent CC′ associated with CDN P cleaves H_aa′_ to yield the fragmented strand H_aa′-1_ that stabilized constituent AA′ of CDN L, while constituent BB′ associated with CDN L cleaves hairpin H_dd′_ to yield the fragmented product H_dd′-1_ that stabilizes constituent DD′ of CDN P. As a result, the intercommunication of the two networks leads to dynamic temporal feedback-driven reconfiguration of the two CDNs, where the biocatalytic cascade GOx/HRP associated with the constituent AA′, being part of CDN L, reveals a temporal feedback-driven increase in its effectiveness of the GOx/HRP cascade (time-dependent increase in the catalytic rate of generated ABTS^•-^) (Figure 3B), and a feedback-driven increase in the content of constituent DD′ in CDN P and an enhancement in the effectiveness of the ADH/NAD^+^ cascade (time-dependent increase in the catalytic rate of generating NADH, monitored by the NADH-mediated reduction of methylene blue, MB^+^ to MBH) (Figure 3C).

**Figure 4:**
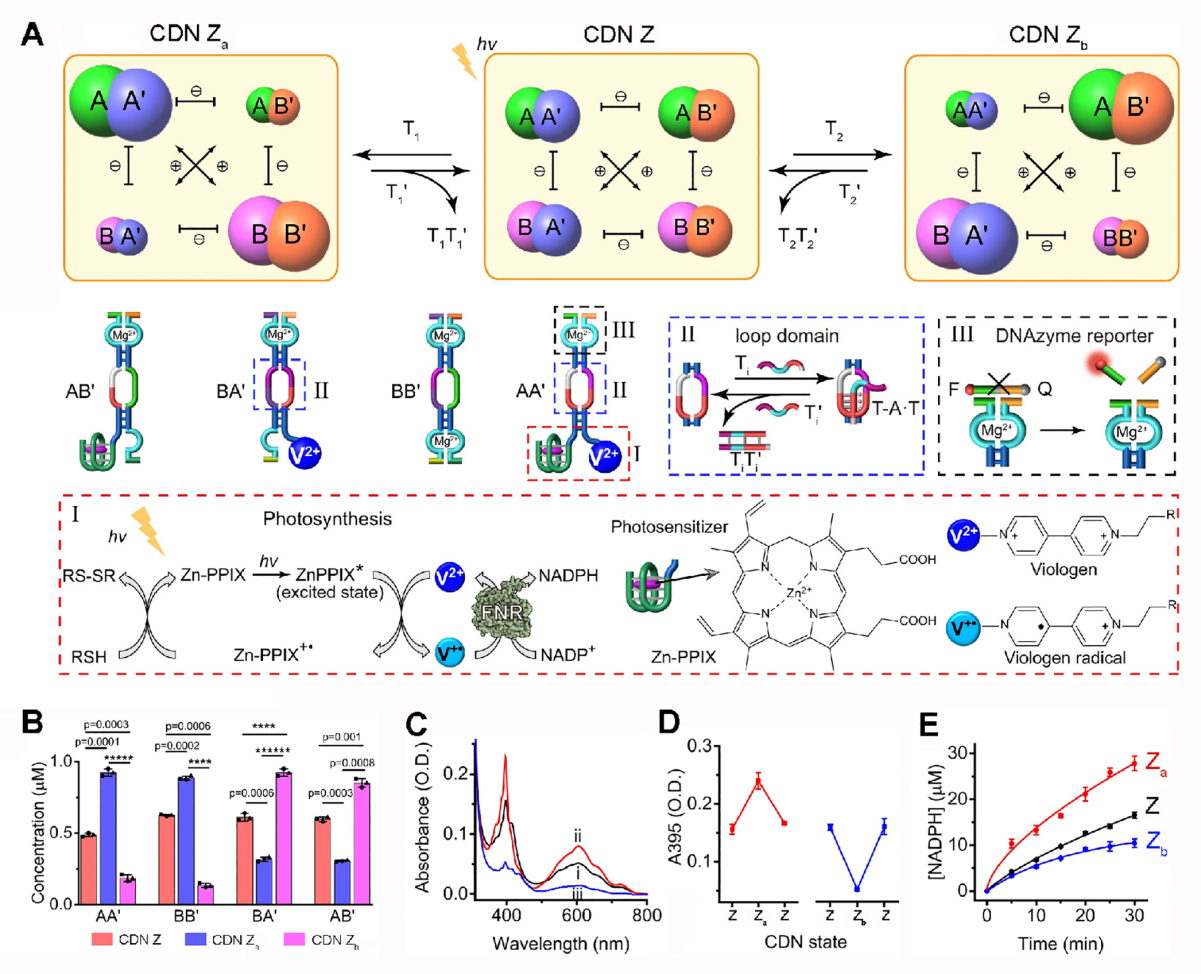
Assembly and operation of an artificial photosynthetic system driven by constitutional dynamic networks: (A) Composition and triggered reconfiguration of the artificial photosynthetic constitutional dynamic network. The artificial photosynthetic process corresponding to the photosensitized generation of the bipyridines radical cation, V^+•^, and the subsequent FNR catalyzed reduction of NADP^+^ to NADPH are displayed in panel I. The reversible reconfiguration processes of the CDNs are stimulated by triggers T_1_/T_1_′ or T_2_/T_2_′ generation or separation of triplex domains in the constituents, panel II; the contents of the constituents in the different CDNs are transduced by the Mg^2+^-ion-dependent DNAzyme reported units associated with the constituents, panel III. (B) Concentration of the constituents in CDNs, Z, Z_a_, and Z_b_ transduced by the Mg^2+^-ion-dependent DNAzyme reported units. (C) Absorption spectra corresponding to the V^+•^ generated by the equilibrated reconfigured CDNs: (i) CDN Z, (ii) CDN Z_a_, (iii) CDN Z_b_. (D) Cyclic and reversible CDNs-guided operation of the artificial photosynthetic network monitored by the light-generated V^+•^. (E) Time-dependent concentrations of NADPH generated by the artificial photosynthetic cascades guided by CDNs Z, Z_a_, and Z_b_. Figure adapted from ref (102) with permission (Creative Commons, CC BY 4.0).

The dynamic control of chemical reactions by means of CDNs was extended to include CDN-guided photocatalytic processes, and particularly artificial photosynthetic cascades.^102^Figure 4A outlines the photoactive constitutional dynamic network acting as the artificial photosynthetic reaction module. The network, CDN Z, includes four constituents AA′, BB′, AB′, and BA′, where the components A and A′ composing constituent AA′ are modified with the Zn(II)-protoporphyrin IX, Zn(II)-PPIX, loaded photosensitizer, and the *N*,*N*′-dialkyl-4,4′-bipyridinium, V^2+^, electron acceptor. Each of the constituent in CDN Z includes a biloop region that can be stabilized by an auxiliary strand, T_1_, through the formation of a T-A•T triplex. In addition, each of the constituents is functionalized with a different Mg^2+^-ion-dependent DNAzyme unit that provides a catalytic label that reports the contents (concentrations) of the respective constituents via the DNAzyme catalyzed cleavage of the respective fluorophore/quencher-modified substrates and the use of appropriate calibration curves. Subjecting CDN Z to trigger T_1_ stabilizes constituent AA′, by forming a T-A•T triplex structure in the biloop domain, resulting in the reconfiguration of CDN Z to CDN Z_a_ where AA′ is upregulated, AB′ and BA′ are downregulated, and concomitantly BB′ is upregulated. Treatment of CDN Z_a_ with the counter trigger T_1_′ separates the triplex structure, thereby restoring CDN Z. Similarly, subjecting CDN Z to trigger T_2_ stabilizes BA′ and reconfigures CDN Z to CDN Z_b_ where constituents BA′ and AB′ are upregulated and the contents of constituents AA′ and BB′ are downregulated. Subjecting CDN Z_b_ to the counter trigger T_2_′ restores the parent CDN Z. The concentrations of the constituents in the three CDNs are transduced by the Mg^2+^-ion-dependent DNAzymes reporter units and displayed in Figure 4B. The functionalization of constituent AA′ in the different CDNs with the Zn(II)-PPIX photosensitizer and bipyridinium (V^2+^) electron acceptor units leads to the photoinduced electron transfer within the photosensitizer/electron acceptor pair resulting in Zn(II)-PPIX^+•^ and V^+•^ and the subsequent oxidation of Zn(II)-PPIX^+•^ by sacrificial electron donor (RSH) to the disulfide (RSSR) product (Figure 4A, panel I). The resulting photogenerated bipyridinium radical cation mediates in the ferredoxin NADP reductase, FNR, catalyzed reduction of NADP^+^ to NADPH. The dynamic control over the content of the photoactive photosensitizer/electron acceptor diad in the respective CDNs controls the effectiveness of the biocatalyzed generated NADPH and the switchable reconfigurations of the CDNs lead to switchable photobiocatalytic transformations. Figure 4C shows the absorbance spectra of the V^+•^ generated in the CDNs Z, Z_a_, and Z_b_ under steady-state illumination of the systems. The concentration of generated V^+•^ is dictated by the contentment of constituent AA′. Figure 4D depicts the switchable generation of CDNs-dictated V^+•^ by the different reconfigured CDNs. Figure 4E depicts the time-dependent generation of NADPH guided by the different CDNs. The most effective generation of NADPH is demonstrated by CDN Z_a_, while the least effective photocatalyzed generation of NADPH is guided by CDN Z_b_.

### 2.2 Biocatalytic Cascades Driven by Transient, Dissipative, Out-of-Equilibrium Systems

**Figure 5:**
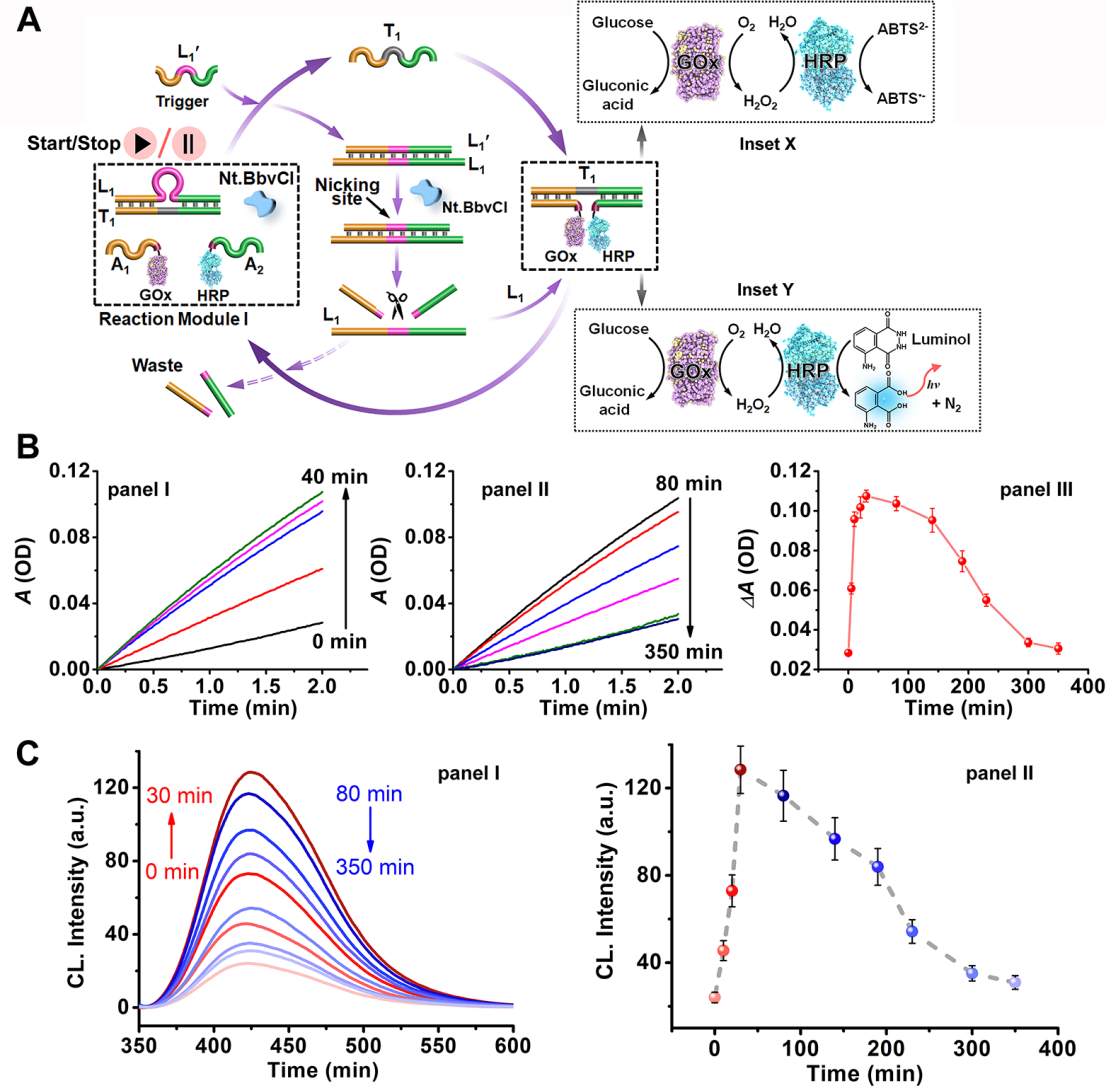
(A) Schematic operation of a nucleic acid-driven dynamic, dissipative, transient glucose oxidase (GOx)/horseradish (HRP) biocatalytic cascade. The bocatalytic cascade involves the aerobic GOx catalyzed oxidation of glucose to gluconic acid and H_2_O_2_ and the subsequent HRP-catalyzed oxidation of ABTS^2–^ to ABTS^•-^, Inset X, or the aerobic GOx catalyzed oxidation of glucose to gluconic acid and H_2_O_2_ and the cascaded HRP catalyzed generation of chemiluminescence in the presence of luminol/H_2_O_2_, Inset Y. (B) Time-dependent transient rates corresponding to the oxidation of ABTS^2–^ to ABTS^•-^ by the GOx/HRP biocatalytic cascade associated with temporal samples withdrawn from the dynamic, transient, reaction module shown in (A). Panel I: Samples corresponding to the temporal buildup of the transient T_1_/A_1_-GOx + A_2_-HRP. Panel II: Samples corresponding to the temporal depletion of the T_1_/A_1_-GOx + A_2_-HRP intermediate. Panel III: Transient dissipative absorbance changes of ABTS^•-^ associated with the temporal build-up and depletion of the biocatalytic intermediate T_1_/A_1_-GOx + A_2_-HRP. (C) Panel I: Chemiluminescence spectra generated by the GOx/HRP biocatalytic cascade in the presence of luminol/H_2_O_2_, using samples withdrawn at time intervals from the dissipative reaction module shown in (A). Panel II: Temporal chemiluminescence intensities generated upon the transient build-up and depletion of the intermediate T_1_/A_1_-GOx + A_2_-HRP bicatalytic supramolecular complex. Figure adapted from ref (103) with permission (Creative Commons, CC BY-NC).

The systems discussed so far present dynamic DNA network-guided biocatalytic processes operating under thermodynamically equilibrated conditions. Biological processes such as cell proliferation and cell motility, or gene regulating networks, operate often under transient, out-of-equilibrium, conditions. The operation of such systems requires the tailoring of stimuli-responsive systems that yield dynamic transient intermediate functionalities while consuming energy supplied by chemical fuels or light/electrical inputs. The information and functionalities encoded in the base-sequence of nucleic acids were used to develop dynamic networks that activate transient, out-of-equilibrium biocatalytic cascades.^103^Figure 5A depicts such dynamic DNA-based transient biocatalytic reaction module. The remaining reaction module includes a duplex L_1_/T_1_, two nucleic acid enzyme conjugates composed of strand A_1_ covalently tethered to glucose oxidase, GOx, A_1_-GOx, and an oligonucleotide strand A_2_ covalently linked to horseradish peroxidase, HRP, A_2_-HRP, and the nicking enzyme Nt.BbvCI. Subjecting the reaction module to the trigger L_1_′ results in the displacement of the duplex L_1_/T_1_ to yield the energetically stabilized duplex L_1_′/L_1_ and the released T_1_ hybridizes with the strands A_1_-GOx and A_2_-HRP to generate the transient intermediate biocatalytic assembly T_1_/A_1_-GOx + A_2_-HRP. The resulting assembly catalyzes the biocatalytic cascade GOx/HRP, where GOx catalyzes the aerobic oxidation of glucose to form gluconic acid and H_2_O_2_ that acts as a substrate for HRP catalyzing oxidation of ABTS^2–^ to the colored product ABTS^•-^. Alternatively, the biocatalytic assembly T_1_/A_1_-GOx + A_2_-HRP activates the biocatalytic cascade where the aerobic oxidation of glucose leads to the coupled HRP-catalyzed generation of chemiluminescence through the oxidation of luminol (Figure 5A, inset Y). The duplex L_1_′/L_1_ was engineered, however, to be nicked by Nt.BbvCI, and the cleaved strand L_1_′ yielded fragmented “waste” products that were separated from L_1_. The released L_1_ displaces T_1_ from the intermediate product T_1_/A_1_-GOx + A_2_-HRP to form the energetically stabilized complex L_1_/T_1_ that restores the parent biocatalytic module. That is, the L_1_′-triggered activation of the reaction module leads to the transient formation of the intermediate biocatalytic GOx/HRP system that catalyzes the transient generation of ABTS^•-^ or chemiluminescence (λ = 425 nm). Figure 5B, panels I and II, shows the time-dependent absorbance changes of the ABTS^•-^ generated by the GOx/HRP bicatalytic cascade stimulated by the intermediate T_1_/A_1_-GOx + A_2_-HRP intermediate generated upon the transient operation depicted in Figure 5A. The contents of the biocatalyst conjugate increase and reach a saturation value within a time interval of ca. 40 min, reflected by an increase in the rates of ABTS^•-^ formation (Figure 5B, panel I) and then the rates of ABTS^•-^ formation in samples of the reaction medium decrease along a time interval of ca. 350 min, consistent with the transient depletion of the T_1_/A_1_-GOx + A_2_-HRP in the system and the recovery of the parent reaction module (Figure 5B, panel II). The transient formation and depletion of the intermediate T_1_/A_1_-GOx + A_2_-HRP are followed by the temporal absorbance of ABTS^•-^ generated by the biocatalyst intermediate in the system (Figure 5B, panel III). Similarly, Figure 5C, panel I, depicts the chemiluminescence spectra generated by the intermediate T_1_/A_1_-GOx + A_2_-HRP cascade reaction along the temporal transient operation of the network. The transient chemiluminescence intensities generated upon the formation and depletion of biocatalyst intermediate are displayed in Figure 5C, panel II. An analog biocatalytic reaction module leading to the L_1_′-triggered transient operation of a lactate dehydrogenase (LDH)/NAD^+^ cascade yielding the transient synthesis of pyruvic acid/NADH products and the coupled transient NADH-mediated reductive amination of pyruvate to l-alanine were demonstrated.

### 2.3 Switchable Dynamic Biocatalysis Driven by DNA Devices

Signal-triggered reconfiguration of nucleic acid nanostructures established versatile means to develop DNA switches and machines, such as tweezers,^18,19,104^ walkers,^20−22^ cranes,^26^ or swinging rings (catenanes or rotaxane).^23,105,106^ Different stimuli, such as fuel/antifuel strand displacement, pH, metal-ion/ligand, or light, have been widely applied as triggers to dynamically modulate the mechanical reconfiguration of DNA nanostructures. In addition, the base sequences of nucleic acids provide instructive information guiding the precise positioning of nucleic acid on DNA scaffolds through dictated hybridization patterns. In particular, the versatile conjugation chemistries allowing the covalent coupling of low-molecular-weight ligands or macromolecules (*e.g.,* proteins) to nucleic acids provide general means to precisely position spatially programmed assemblies of molecular or macromolecular systems on nucleic acid scaffolds. That is, the hybridization of nucleic acid–protein conjugates with pre-engineered protruding strands associated with DNA scaffolds yields programmed, spatially ordered arrays on the DNA scaffolds. Furthermore, besides the organization of functional assemblies on one-dimensional DNA scaffolds, the programmability of nucleic acid structures allows the assembly of two-dimensional nanostructures, such as Y-shaped,^107^ crossover tetra-armed shaped structures^108^ or self-assembled complex two-dimensional origami structures,^109^ and even the functionalization of three-dimensional DNA structures, such as DNA tetrahedra, DNA origami “boxes”,^110^ or capsules.^111^ These diverse structures enable the dictated spatial positioning of DNA-modified functionalities not only on the surfaces of the nanostructures but also at geometrically engineered edge positions of the supports. The integration of mechanically switchable properties into the DNA frameworks that are modified with spatially engineered catalytic sites is then anticipated to yield dynamic mechanically switchable catalytic assemblies and to intercommunicated switchable catalytic cascades in spatially confined environments. This section will dynamically exemplify such reconfigurable catalytic devices.

**Figure 6:**
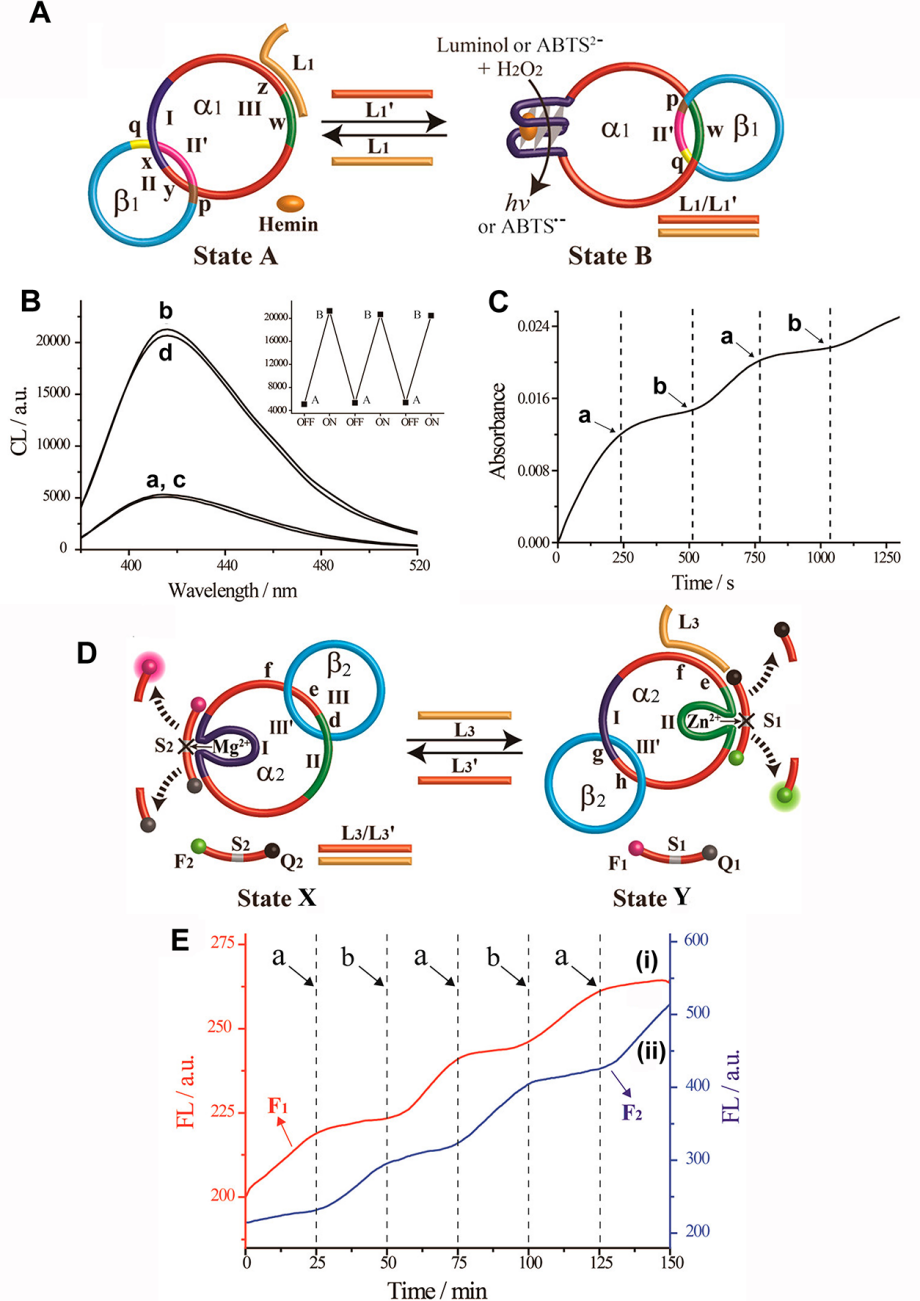
(A) Dynamic reversible and cyclic reconfiguration of an interlocked DNA two-ring catenane system using fuel/antifuel strands as triggers. The reconfiguration of the catenane in state A to state B yields a hemin/G-quadruplex DNAzyme unit in ring α1 that stimulates the hemin/G-quadruplex catalyzed generation of chemiluminescence in the presence of luminol/H_2_O_2_ or the catalyzed oxidation of ABTS^2–^ by H_2_O_2_ to form the colored ABTS^•-^. (B) Chemiluminescence spectra generated by curves (a) and (c)—state A, curves (b) and (d)—state B, and inset—cyclic chemiluminescence responses of the system. (C) Switchable absorbance chances of ABTS^•-^ generated upon the cyclic reconfiguration of the catenane between states B and A. At times marked (a), the system is switched to state A, and at times marked (b), the molecular device is switched to state B. (D) Dynamic reversible reconfiguration and switching of an interlocked two-ring catenane between state X and state Y, using fuel/antifuel strand displacement triggers, that activate switchable Mg^2+^-ion-dependent DNAzyme and Zn^2+^-ion-dependent DNAzyme functions. (E) (i) Switchable catalytic activities of the Zn^2+^-ion-dependent DNAzyme; (ii) switchable catalytic activities of the Mg^2+^-ion-dependent DNAzyme. At times marked with arrows (a), L_3_′ is added to yield state X, and at times marked with (b), L_3_ is added to yield state Y. Figure adapted from ref (112) with permission. Copyright 2015, American Chemical Society.

Figure 6A exemplifies a supramolecular interlocked two-ring catenane DNA device for the switchable dynamic operation of a hemin/G-quadruplex catalyst.^112^ The interlocked structure consists of two rings α1 and β1 where ring α1 includes sequence II hybridized with sequence II′ that is a part of ring β1. In addition, ring α1 includes sequence III that is composed of two subsequences z and w, that are blocked, through hybridization, with an auxiliary strand L_1_. The sequence I is composed of the G-rich sequence that is caged in the catenated structure, and domain II′ of ring β1 includes two subsequences p and q that are complementary to the sequence w caged in the duplex III/L_1_ structure. The displacement of strand L_1_ by a fuel strand L_1_′ yields the duplex L_1_/L_1_′, resulting in the “bare” sequence w that allows the dynamic transition of ring β1 to domain w yielding the energetically stabilized duplex between domains p/q and w. The transition of ring β1 to site w associated with ring α releases the G-rich sequence I associated with ring α1, resulting in switched-on catalyzed oxidation of ABTS^2–^ by H_2_O_2_ to the colored product ABTS^•-^, in the presence of K^+^-ions, hemin, and the reconfigured sequence I into the hemin/G-quadruplex DNAzyme, state B. Subjecting state B to the trigger L_1_ displaces ring β1 from the sequence w, the L_1_-locked sequence z/w, and the release of ring β1 that dynamically restores state A, where domain II′ in ring β1 hybridizes to domain II associated with ring α1. This leads to dissociation of the G-quadruplex structure and to switching off the catalytic activity of the DNAzyme. By the cyclic activation of the catenated device with triggers L_1_′/L_1_, the catalytic functions of the device were dynamically switched between “ON” and “OFF” states, that were followed by the temporal formation and blockage of the colored ABTS^•-^ products (Figure 6B and C). In a related system, a two-ring catenane system was designed to switch catalytic activities between two DNAzymes (Figure 6D). In state X, the Mg^2+^-ion-dependent DNAzyme consisting of two catenated rings α2 and β2 is activated, and it cleaves the fluorophore F_2_ (FAM)-quencher Q_2_ (Iowa black FQ)-modified substrate S_2_ to yield the fluorescent readout signal. In state X, the hybridization of domains III and III′ of rings α2 and β2 blocks a part of the Zn^2+^-ion-dependent DNAzyme sequence encoded in the rim of ring α2. Treatment of the two-ring catenane in state X with the fuel strand L_3_ results in the displacement of ring β2 and its hybridization to the domains (g+h) of α2, while unlocking domain III is associated with ring α2. The released ring α2 forms an energetically stabilized interlocked hybrid duplex III′/(g+h) catenated structure, and concomitantly the released sequence II reorganizes into the Zn^2+^-ion-dependent DNAzyme, state Y, that cleaves the F_1_ (ROX)/quencher (Q_1_ = BHQ2)-modified substrate S_1_ to yield the fluorescent F_1_-fragment. Subjecting state Y to the counter fuel strand, L_3_′, reconfigures state Y into state X. That is, by the cyclic treatment of the α2/β2 two-ring interlocked catenane, the switchable dynamic transitions between state X and state Y lead to the dynamic, orthogonally operating, DNAzymes (the Mg^2+^-ion-dependent DNAzyme and the Zn^2+^-ion-dependent DNAzyme) (Figure 6E).

**Figure 7:**
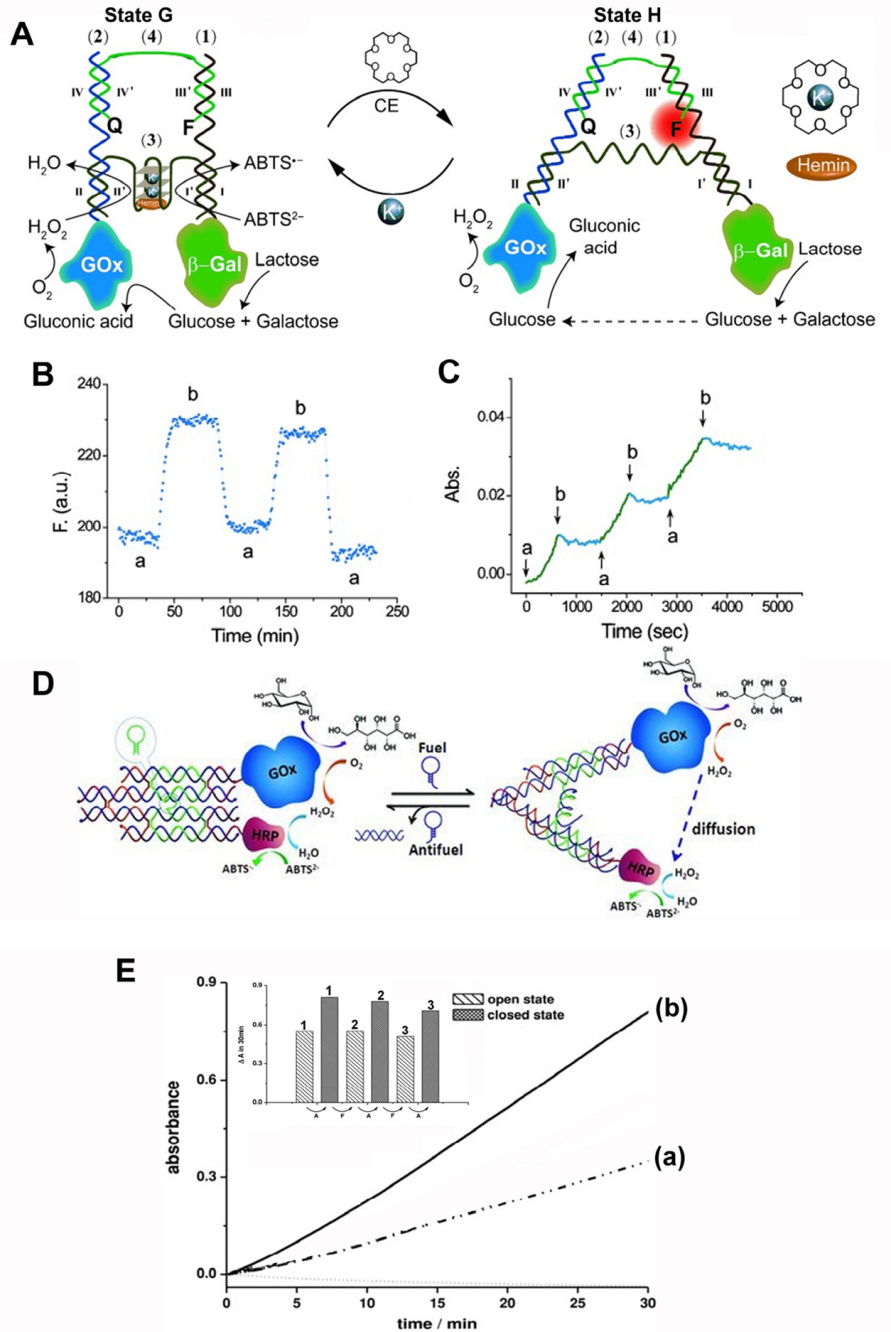
(A) Schematic operation of a tweezers-like biocatalytic conjugate consisting of three biocatalysts β-Gal/GOx/hemin-G-quadruplex using 18-crown-6-ether and K^+^-ions as reconfiguration triggers. The biocatalytic cascade involves the β-Gal catalyzed hydrolysis of lactose to yield galactose and glucose, the subsequent GOx-catalyzed aerobic oxidation of glucose to gluconic acid and H_2_O_2_, and the subsequent hemin/G-quadruplex-catalyzed oxidation of ABTS^2–^ by H_2_O_2_ to the colored ABTS^•-^ product. The “closed” state of the tweezers, state G, is reconfigured through separation of the G-quadruplex by 18-crown-6-ether, into the “open” tweezer configuration, state H. The “open” tweezers, state H, is reconfigured into state G upon addition of K^+^-ions that bridges the tweezers arm by a K^+^-ion stabilized hemin/G-quadruplex stabilized unit. The tweezers arms are modified with a fluorophore (Cy 5.5)/quencher (Iowa Black RQ) pair that transduces the mechanical opening/closing of the tweezers by the fluorescence response of the fluorophore/quencher pair. (B) Switchable fluorescence changes upon the cyclic “mechanical” opening and closing of the tweezers, in the presence of added crown-ether/K^+^-ions, respectively. (C) Time-dependent absorbance change of ABTS^•-^ upon the cyclic “ON” and “OFF” activation of the three-catalyst β-Gal/GOx/hemin-G-quadruplex cascade. At times marked with (a), the K^+^-ion-stabilized β-Gal/GOx/hemin-G-quadruplex “closed” tweezers structure, state G, is formed, and at times marked with (b), the crown-ether stimulated opening of the tweezers proceeds to yield the switched “OFF” biocatalytic cascade, state H. (D) Dynamic switching of the GOx/HRP biocatalytic cascade using a double-cross over DNA structure locked by an immobile four-way junction. The device is switched between “open” and “closed” states by applying fuel/antifuel strands. (E) Time-dependent absorbance changes of ABTS^•-^ generated by the GOx/HRP bienzyme cascade in the presence of (a) “open” tweezer structure; (b) “closed” tweezer structure. Inset: Reversible operation of the GOx/HRP cascade by the tweezers device. Figure 7A–C adapted from ref (113) with permission. Copyright 2014 John Wiley and Sons. Figure 7D and E adapted from ref (117) with permission. Copyright 2014 John Wiley and Sons.

A three-catalyst cascade^113^ operating by a dynamic tweezer device is exemplified in Figure 7A. The oligonucleotide–protein conjugates (**1**)-functionalized β-galactosidase, (**1**)-β-Gal, and (**2**)-modified GOx, (**2**)-GOx, are cross-linked by a fluorophore (F = Cy5.5)/quencher (Q = Iowa Black RQ) modified strand (**4**) to yield a supramolecular three-catalyst nanostructure. In addition, the two nucleic acid conjugates consisting of (**1**)-β-Gal and (**2**)-GOx were bridged by a nucleic acid (**3**) that self-assembles into a hemin/G-quadruplex DNAzyme cross-linking unit, in the presence of K^+^-ions and hemin, state G. The bifunctional cross-linked structure in state G activates the three-catalyst cascade where β-Gal hydrolyzes lactose to galactose and glucose. The resulting glucose is aerobically oxidized to gluconic acid and H_2_O_2_ by GOx, and the formed H_2_O_2_ provides the oxidant for the hemin/G-quadruplex-catalyzed oxidation of ABTS^2–^ to the colored ABTS^•-^ that provides the transduction signal for the three-catalyst cascade. Treatment of state G with 18-crown-6-ether separates the bridging hemin/G-quadruplex structure, resulting in an extended opened configuration of the tweezers, state H, in which the three-catalyst cascade is blocked. Readdition of K^+^-ions to the system restores the closed hemin/G-quadruplex-driven tweezers in which the three-catalyst cascade is reactivated. By the cyclic addition of 18-crown-6-ether and K^+^-ions, the dynamic switching of the three-catalyst cascade between ON–OFF states was demonstrated (Figure 7B). The dynamic transitions of the tweezers device between closed and open configurations were followed by probing the fluorescence responses of the fluorophore/quencher-functionalized bridging unit (Figure 7C). While the fluorophore is effectively quenched in the closed configuration, state G, the fluorescence of the probe is intensified in the open configuration, state H, where spatial separation between the fluorophore and quencher exists. It should be noted, however, that although the hemin/G-quadruplex is a useful switchable DNAzyme probe in operating biocatalytic cascades, its long-term participation in cyclic cascade is limited due to its reconfiguration into different G-quadruplex motifs, revealing alternate catalytic activities.^114^ Related studies have applied dynamic tweezers structures to operate switchable biocatalytic cascades.^115−117^ For example,^117^ a double crossover (DX) rigid structure consisting of two arms, modified at each of their ends with GOx and horseradish peroxidase (HRP), bridged by an immobile joint to yield the open tweezers structure. By the reverse displacement of the fuel strand with an antifuel hairpin, the rigid joint double crossover structure was restored. As in the condensed rigid structure, where the two enzymes GOx and HRP are in intimate contact, the bienzyme cascade whereby the aerobic GOx catalyzed oxidation of glucose to gluconic acid and H_2_O_2_ proceeds, followed by the effective channeling of H_2_O_2_ to the neighboring HRP that catalyzed the H_2_O_2_ oxidation of ABTS^2–^ to the colored product ABTS^•-^ (Figure 7D). The fueled opening of the closed structure into the open tweezers state spatially separate the two enzymes perturbing the bienzyme cascade. By the reversibly opening and closing of the tweezers structure the biocatalytic cascade was switched between low and high activity (Figure 7E). The similar concept was adapted to apply the dynamic opening and closing to switch the glucose-6-phosphate dehydrogenase/NAD^+^ biocatalytic cascade.^116^ Furthermore, the dynamic switchable control of enzymes was demonstrated by designing nucleic acid programmed enzyme–inhibitor scaffolds where the intimate inhibition of the enzyme is stimulated by strand displacement or light-induced control over the distance separating the enzyme/inhibitor constituents.^118−120^

### 2.4 Dynamic Switchable Biocatalysis on DNA Frameworks

**Figure 8:**
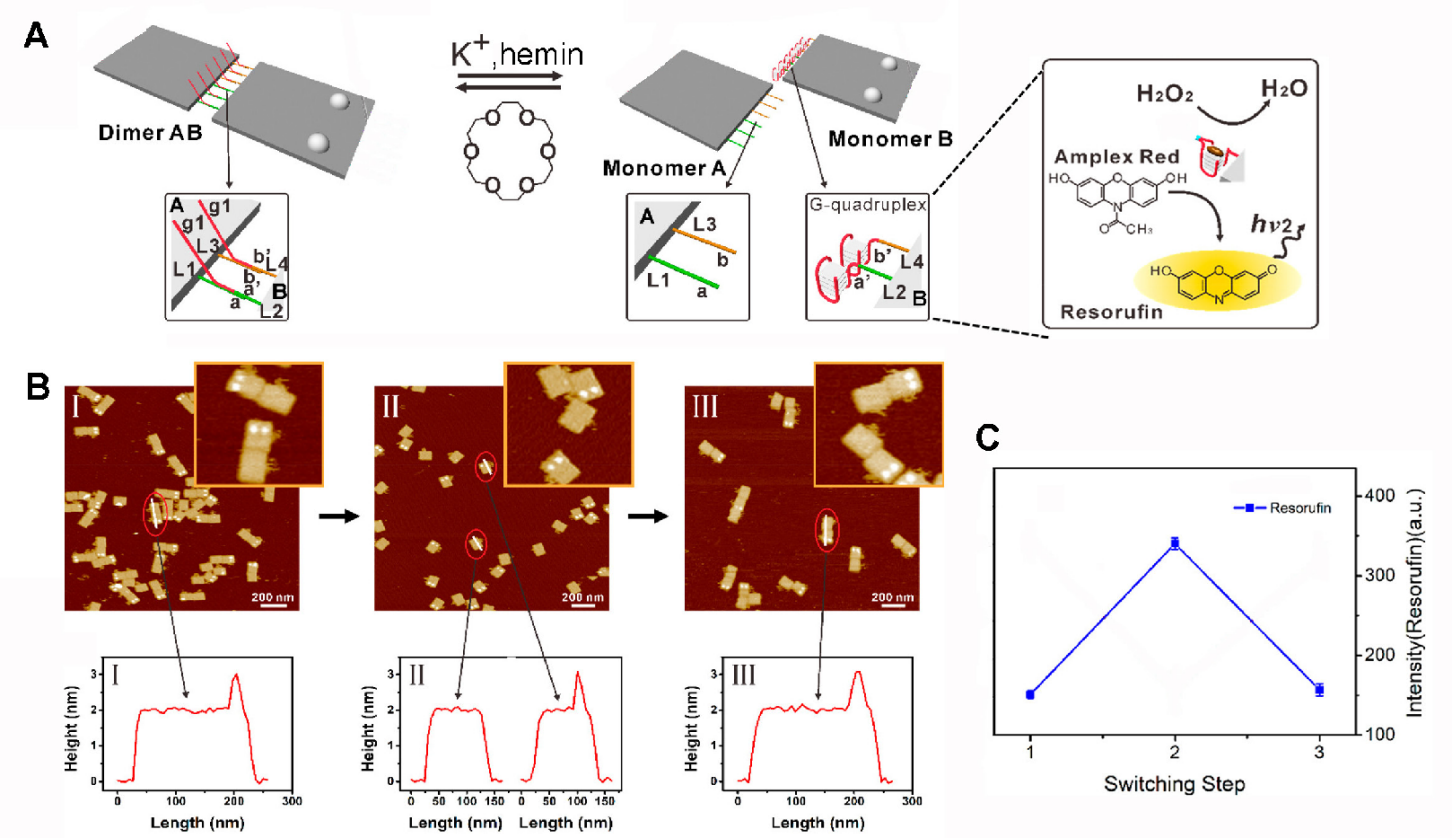
(A) Schematically switchable dimerization and separation of two origami tiles using hemin/G-quadruplex DNAzyme bridges, triggered by K^+^-ions and 18-crown-6-ether. Inset: DNAzyme catalyzes the Amplex-Red oxidation of by H_2_O_2_ to the fluorescent Resorufin. (B) AFM images and cross section analysis corresponding to the dimerized, panel I; K^+^-ion-separated tiles, panel II; and crown-ether redimerized tiles, panel III. (C) Switchable catalyzed activation of the hemin/G-quadruplex DNAzyme upon the triggered separation and dimerization of the origami tiles. Figure adapted from ref (121) with permission. Copyright 2018 American Chemical Society.

The spatial positioning of catalytic units on two-dimensional DNA frameworks provides a further means to dynamically control catalytic functions of biomolecular conjugates in organized nanoenvironments and to guide programmed biocatalytically driven cascades. DNA origami scaffolds provide a remarkable structure to precisely position and dynamically activate catalytic assemblies and particularly switchable catalytic systems. The assembly of origami rafts or tiles by a programmed set of “stapler” units that links together along circular DNA enables the integration of functional (catalytic) nucleic acids at the edges of the origami tiles or the functionalization of predesigned positions of the upper or lower origami surfaces by the extension of stapler units with protruding tethers to which nucleic acid-modified catalyst are anchored. Figure 8A exemplifies the switchable dynamic separation and formation of an origami dimer structure that leads to the reversible assembly of hemin/G-quadruplex units at the edge of the origami-tiles.^121^ The origami dimer consists of two tiles A and B interbridged by complementary nucleic acids L_1_/L_2_ and L_3_/L_4_ integrated at the edges of the origami tiles A and B (marked with two “dots” composed of 3 × surface-tethered hairpins). The nucleic acids L_2_ and L_3_ include guanosine-rich tethers g_1_ partially caged in the duplex a/a′ and b/b′ domains bridging the duplexes L_1_/L_2_ and L_3_/L_4_. Treatment of the AB dimers with K^+^-ions separates the dimers while self-assembling the tethers L_2_, L_4_ into K^+^-stabilized G-quadruplexes linked to the edge of tile B. In the presence of hemin, the resulting hemin/G-quadruplex units provide catalytic units for the H_2_O_2_-catalyzed oxidation of Amplex Red to the fluorescent Resorufin (Figure 8A, inset). Subjecting the separated origami tiles to 18-crown-6-ether dissociates the hemin/G-quadruplex units leading to the energetically stabilized catalytically inactive AB dimers. The switchable separation and reformation of the origami dimers AB was followed by atomic force microscopy (AFM) (Figure 8B), and the dynamic switchable catalytic functions of the hemin/G-quadruplex catalyzed oxidation of Amplex Red were demonstrated (Figure 8C).

**Figure 9:**
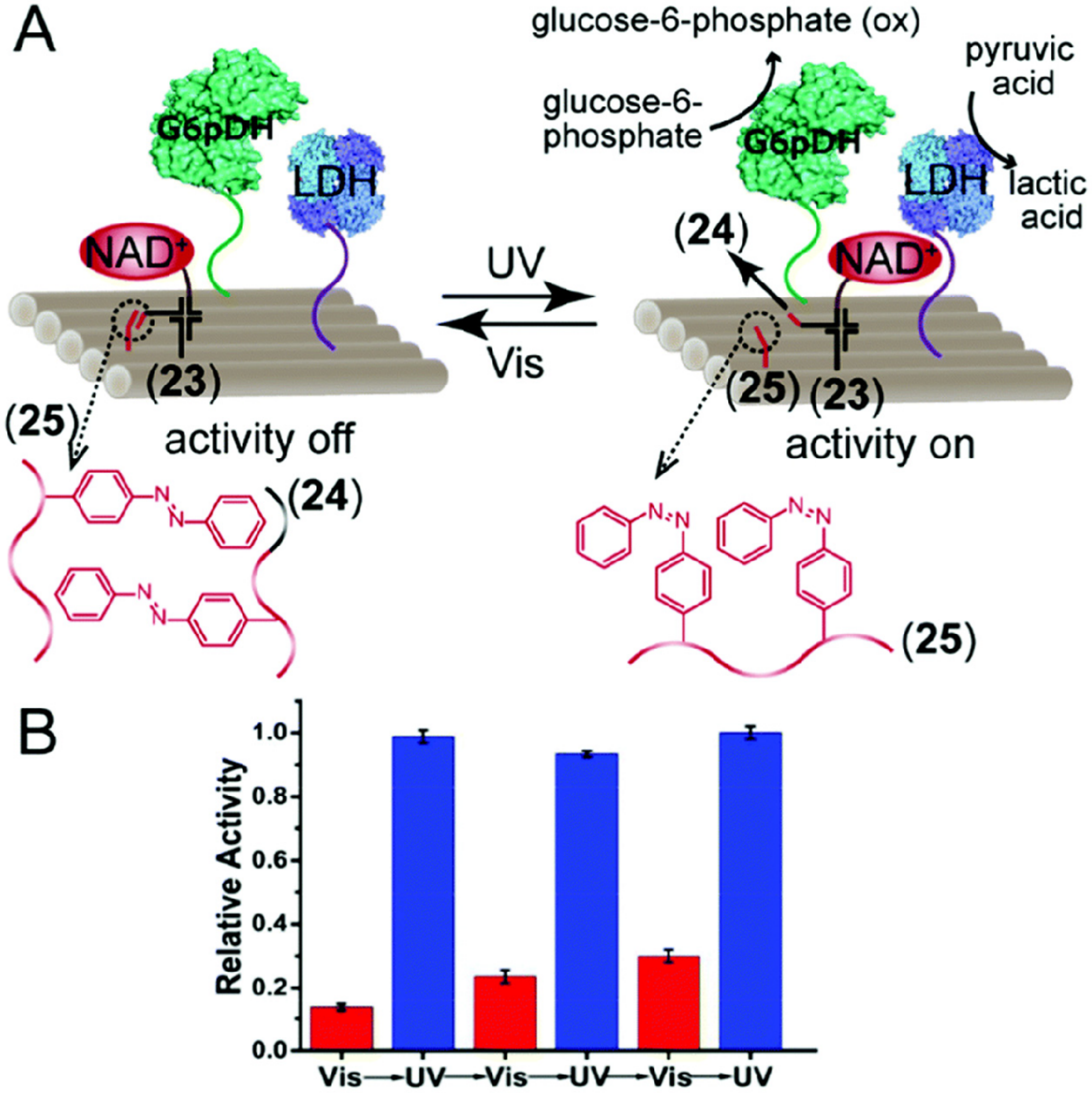
(A) Schematic photoinduced activation of a NAD^+^-cofactor biocatalytic cascade consisting of G6pDH and lactate dehydrogenase (LDH), on a DNA origami tile, using a mediating NAD^+^-cofactor tethered to an azobenzene photoisomerizable foothold associated with the origami tile. (B) Switchable ON/OFF catalytic activities of the photoresponsive NAD^+^-mediated bienzyme cascade: Switching “OFF” in the presence of the *trans*-azobenzene modified foothold (generated upon visible light illumination), switching “ON” in the presence of the *cis*-azobenzene foothold (generated upon UV light illumination). Figure adapted from ref (122) with permission. Copyright 2018 American Chemical Society.

The dynamic light-induced switchable NAD^+^ mediated activation of the biocatalytic cascade between glucose-6-phophate dehydrogenase (G6pDH) and lactate dehydrogenase (LDH) on a two-dimension origami tile is displayed in Figure 9A using a phototriggered NAD^+^-mediated swinging arm.^122^ The enzyme conjugates G6pDH and LDH modified with nucleic acid tethers were positioned in spatially proximate sites on the origami tile. The NAD^+^ cofactor was linked through a Holliday Junction structure (**23**), positioned in between the two enzymes on the tile framework. The Holliday Junction unit includes a swinging arm (**24**) that forms a stable duplex with a *trans*-azobenzene foothold tether (**25**). Formation of the *trans*-azobenzene functionalized (**24**)/(**25**) duplex swings the NAD^+^-cofactor away from the two enzyme components prohibiting the cofactor-induced communication between the biocatalysts. Photoisomerization of the *trans*-azobenzene units to the *cis*-states separates the duplex (**24**)/(**25**) allowing the “mechanical” swinging of the NAD^+^-cofactor unit into spatial proximity to the two biocatalysts. This switches on the G6pDH catalyzed oxidation of glucose-6-phosphate to d-glucono-1,5-lactone 6-phosphate with the concomitant reduction of NAD^+^ to NADH (eq 1). The resulting reduced cofactor mediates the reduction of pyruvic acid to lactic acid (eq 2).
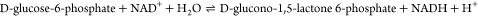
1
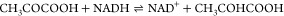
2

Thus, the light-induced swinging of the cofactor into the proximity of the two enzymes switches on the biocatalytic cascade. The reverse photoisomerization of the *cis*-azobenzene units to the *trans-*states swings back the cofactor arm to the inactive state of the origami device. By the cyclic photoisomerization of the azobenzene units between *cis* and *trans* states, the dynamic swinging of the NAD^+^ cofactor arm enabled the “ON” and “OFF” operation of the biocatalytic cascade (Figure 9B).

**Figure 10:**
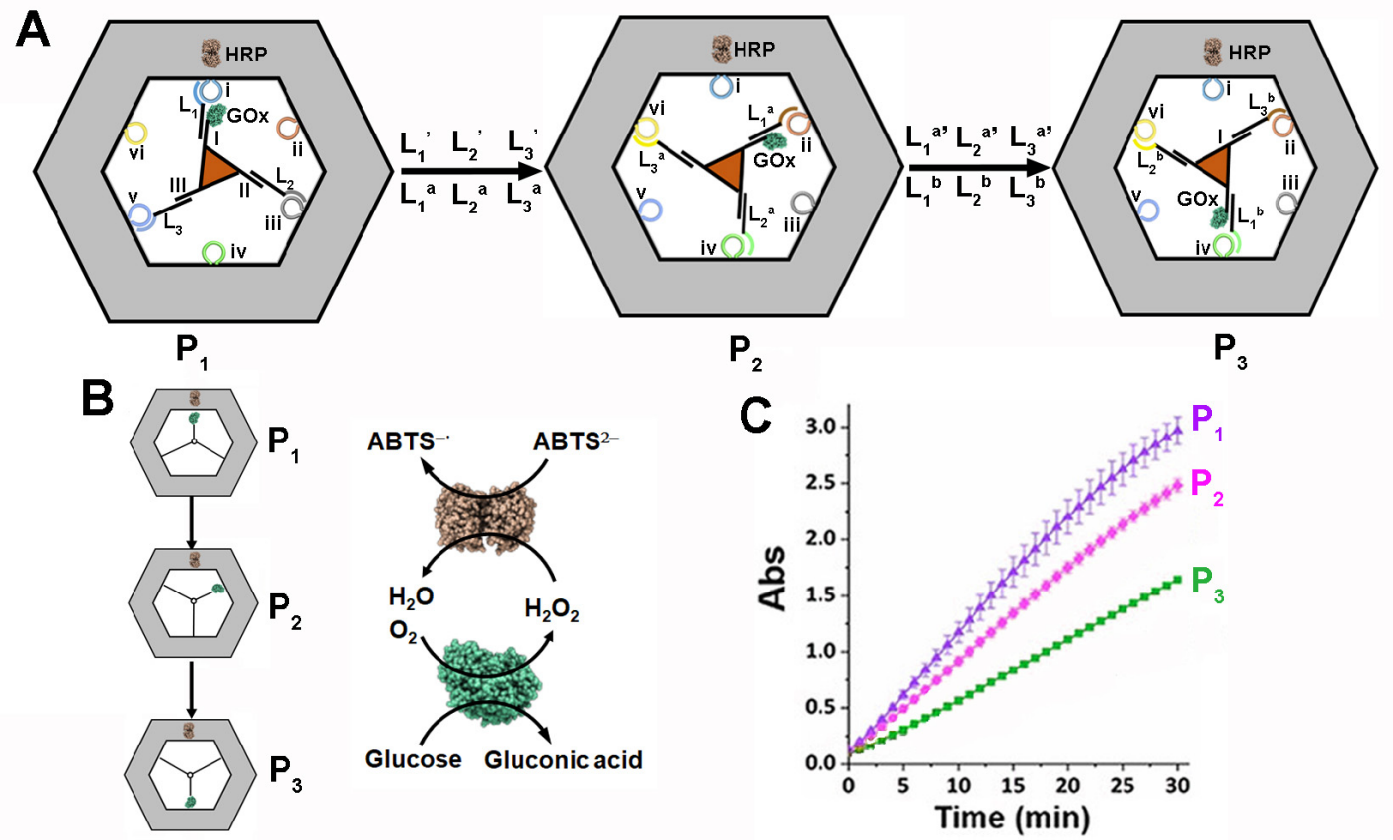
(A) Dynamic control over the glucose oxidase (GOx)/horseradish peroxidase (HRP) cascade by the rotatable transitions of the biocatalytic cascade within an origami framework using the strand displacement principle. (B) Clockwise dynamic transitions of the GOx/HRP cascade across the states P_1_, P_2_, and P_3_. The biocatalytic cascade involves the GOx catalyzed aerobic oxidation of glucose to gluconic acid and H_2_O_2_, followed by the HRP catalyzed oxidation of ABTS^2–^ by H_2_O_2_ to the colored ABTS^•-^ product. (C) Time-dependent absorbance changes corresponding to the GOx/HRP biocatalytic cascade generating ABTS^•-^ by the states P_1_, P_2_, and P_3_. Figure adapted from ref (123) with permission. Copyright 2019 John Wiley and Sons.

The dynamic rotational control over a biocatalytic cascade operating on an origami scaffold consisting of a hexagonal frame was demonstrated^123^ (Figure 10). The six interedges of the frame were functionalized with six hairpin tethers (i)–(vi). The enzyme HRP is firmly positioned on the hexagonal DNA origami surface through hybridization to a protruding tether. A three-arm stator comprising nucleic acid I-linked to the enzyme GOx and nucleic acids II and III was positioned in the void volume of the origami frame by linking the tethers I to (i), II to (iii), and III to (v) through bridging nucleic acids L_1_, L_2_, and L_3_, resulting in state P_1_ (Figure 10A). Subjecting the device in state P_1_ to the displacement strand L_1_′, L_2_′, and L_3_’ and to bridging strand L_1_^a^, L_2_^a^, and L_3_^a^ results in the rotation of the stator to state P_2_, where the stator arms are fixed to tethers (ii), (iv), and (vi) using the bridges L_1_^a^, L_2_^a^, and L_3_^a^. The subsequent treatment of state P_2_ with the counter strands L_1_^a^′, L_2_^a^′, and L_3_^a^′ and the helper strands, L_1_^b^, L_2_^b^, and L_3_^b^ result in a further rotation step to yield state P_3_. These dynamic rotations lead to the control over the spatial separation of the enzymes, GOx and HRP. While in state P_1_ the two biocatalysts are in proximity, in state P_2_ the two biocatalysts are partially spatially separated, and in state P_3_ the biocatalysts are spatially apart. As a result, the biocatalytic cascade, consisting of the GOx-mediated aerobic oxidation of glucose to gluconic acid and H_2_O_2_ and the subsquent HRP catalyzed oxidation of ABTS^2–^ to ABTS^•-^ by H_2_O_2_ is most effective in state P_1_, and it gradually decreases to lower activity in state P_2_ and lowest activity in state P_3_ (Figure 10B). This is, indeed, reflected by the order of the rates of the biocatalytic cascade generating ABTS^•-^ by the respective states P_1_, P_2_, and P_3_ (Figure 10C).

**Figure 11:**
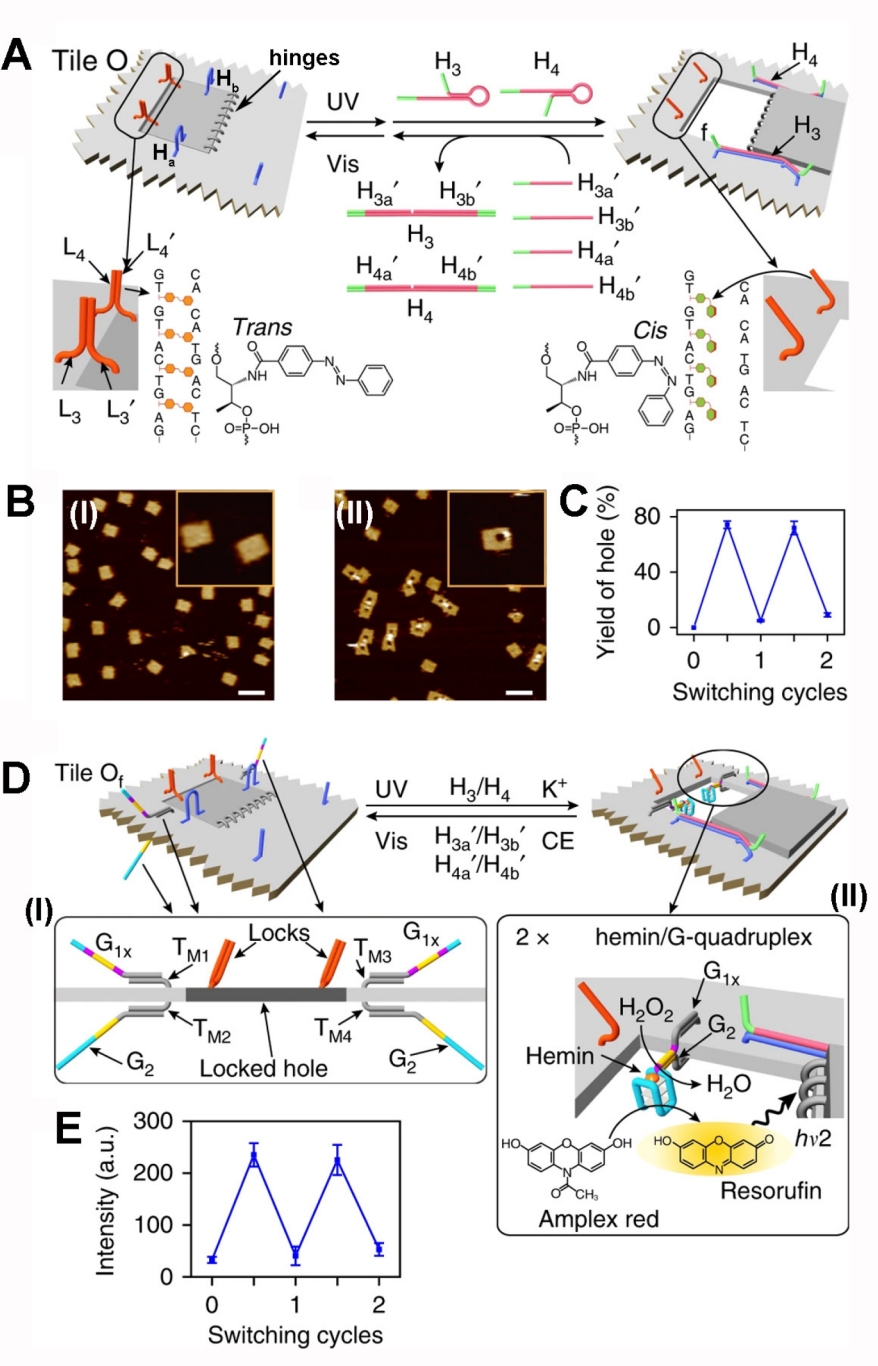
(A) Schematic photoinduced dynamic unlocking and locking of a nanohole in an origami raft using photoisomerizable *trans*/*cis*-azobenzene-modified nucleic acid locks. (B) AFM images corresponding to panel I: the locked nanohole domain associated with the DNA origami rafts; panel II: the photoinduced unlocked nanohole-modified nanohole rafts. (C) Light-induced switchable closed/open nanohole-modified origami rafts. (D) Schematic photoresponsive DNA origami tile that includes a *trans*-azobenzene-locked domain functionalized on opposite faces with engineered G-quadruplex subunit tethers. The photoinduced unlocking of the *trans*-azobenzene locks yields nanoholes that, in the presence of K^+^-ions and hemin, enable the self-assembly of hemin/G-quadruplex DNAzyme units in the confined nanohole environment. The DNAzyme catalyzes the Amplex Red oxidation of by H_2_O_2_ to the fluorescent Resorufin. By the cyclic *cis*/*trans* photoisomerization of locking units, the reversible opening and closing of the nanoholes proceeds, resulting in the switchable ON/OFF biocatalytic functions in the nanohole domain. (E) Switchable ON/OFF hemin/G-quadruplex catalyzed oxidation of Amplex Red by H_2_O_2_ to form fluorescent Resorufin. Figure adapted from ref (124) with permission (Creative Commons, CC BY 4.0).

The dynamic “mechanical” light-induced opening and closing of a nanohole in a DNA origami tile, and the switchable operation of a catalytic reaction in the confined nanohole^124^ are presented in Figure 11. An origami tile 100 nm × 100 nm was engineered to include a surface patch consisting of the two duplex *trans*-azobenzene locks composed of the strands L_3_/L_3_′ and L_4_/L_4_′ associated with the origami raft and patch, respectively, and eight “hinges” linking the patch to the raft (counter to the locking edge). In addition, two handles H_a_/H_b_ on opposite sides of the patch link the patch to the origami raft (Figure 11A). Illumination of the origami tile, λ = 365 nm, in the presence of the hairpins H_3_ and H_4_, results in the photoisomerization of the *trans*-azobenzene units to the *cis*-state, to the separation of the L_3_/L_3_′ and L_4_/L_4_′ locks. The concomitant binding of the hairpins H_3_/H_4_ to the handles H_a_/H_b_ bridging the patch to the raft, assist with the opening of the nanohole on the origami raft by turning the patch across the hinges and its stretching over the raft by linking the opened hairpin through hybridization of H_a_/H_3_ and H_b_/H_4_ to the foothold anchoring sites associated with the origami raft. This mechanical, light-induced opening of the patch yields a nanohole (ca. 50 nm diameter) in the origami raft (Figure 11B). By the reverse photoisomerization of the *cis-*azobenzene units to the *trans*-azobenzene state, and applying the strands H_3a_′, H_3b_′, H_4a_′, and H_4b_′ as antifuel strands that disconnect the stretching units H_3_ and H_4_ from the anchoring footholds and the handles, the parent locked origami device is formed. By the reversible and cyclic light-induced unlocking of the *trans*-azobenzene locks to the *cis*-state and applying the fuel hairpins H_3_/H_4_, the nanoholes were reopened, and by the reverse light-induced isomerization of the photoisomerizable units to the *trans*-state and applying the antifuel strands H_3a_′, H_3b_′, H_4a_′, and H_4b_′, the locked origami structure was regenerated. That is, the cyclic photoisomerization of the *cis*-state origami structure in the presence of the hairpins H_3_, H_4_ and the reverse photoisomerization of the *trans*-azobenzene units, in the presence of H_3a_′, H_3b_′, and H_4a_′, H_4b_′, leads to the dynamic switchable and reversible opening and closing of the nanoholes in the origami rafts (Figure 11C). The further functionalization of the origami raft allows the switchable “ON” and “OFF” operation of the catalytic hemin/G-quadruplex DNAzyme in the nanometer-sized nanoholes, acting as a confined reaction volume for the switchable catalytic process (Figure 11D). Two pairs of guanosine-rich nucleic acid strands, with encoded sequences to act a G-quadruplex subunits, G_1*x*_/G_2,_ were hybridized with appropriate protruding tethers T_M1_, T_M2_, T_M3_, and T_M4_, linked as protruding tethers to the upper surface and counter surface of the origami raft at edges (Figure 11D, inset I). The light-induced unlocking of the nanoholes, in the presence of K^+^-ion and hemin, results in the self-assembly of a two K^+^-ion-stabilized G-quadruplex DNAzyme units catalyzing the oxidation of Amplex Red by H_2_O_2_ to the Resorufin fluorescent product (Figure 11D, inset II). The reverse photoinduced closing of the nanohole, in the presence of 18-crown-6-ether (CE), that separates the G-quadruplexes, results in the catalytic units and the locking of the nanoholes. By the cyclic and reversible light-induced dynamic opening and closing of the nanoholes, in the presence of coadded K^+^-ions/CE, the catalytic process in the nanoholes is switched between “ON”/“OFF” states (Figure 11E). Similar “mechanical” dynamic unlocking of nanoholes in origami rafts and operation of cyclic catalytic reactions in the nanoholes were reported using switchable aptamer-ligand complexes as locks.^125^

**Figure 12:**
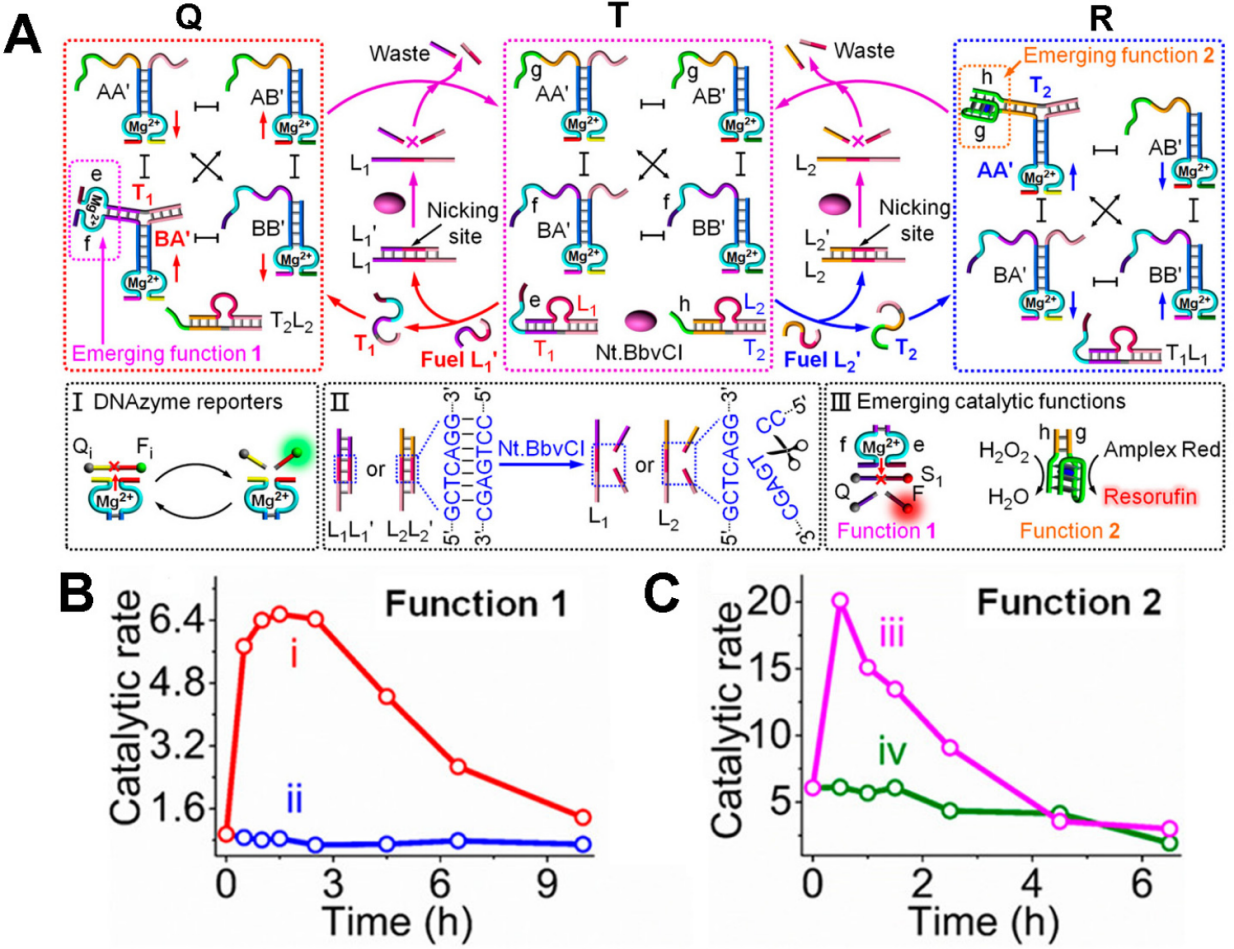
(A) Schematic reaction module consisting of an equilibrated constitutional dynamic network, CDN T, two functional duplexes L_1_/T_1_ and L_2_/T_2_ and nicking enzyme, Nt.BbvCI, that undergoes the L_1_′- or L_2_′-fueled transient transition of CDN T to CDN Q or CDN R and to the dissipative nickase-driven recovery to CDN T. The transient transition of CDN T to Q leads to the emergence of the transient active Mg^2+^-ion-dependent DNAzyme (function 1), whereas the L_2_′-stimulated dynamic transition of CDN T to CDN R yields the emergent transient hemin/G-quadruplex DNAzyme activity (function 2), panel III. (B) Transient catalytic rates of the emergent Mg^2+^-ion-dependent DNAzyme (function 1): upon L_1_′-triggered the temporal transient transition of CDN T to CDN Q, curve (i); upon L_2_′-triggered the temporal reconfiguration of CDN T to CDN R and back, curve (ii). (C) Transient catalytic rates of the emergent hemin/G-quadruplex DNAzyme (function 2): upon L_2_′-triggered the temporal transient transition of CDN T to CDN R and back, curve (iii); upon L_1_′ triggered the temporal reconfiguration of CDN T to CDN Q and back, curve (iv). Adapted from ref (126) with permission. Copyright 2020, American Chemical Society.

The dynamic equilibration of constitutional dynamic networks (CDNs) and the triggered operation of dissipative out-of-equilibrium systems generating transient chemical intermediates were integrated as functional motives to engineer dissipative self-assembled networks revealing dynamic catalytic properties^126^ (Figure 12A). The system consists of a constitutional dynamic network, CDN T, two coadded duplexes L_1_/T_1_ and L_2_/T_2_, and the nicking enzyme, Nt.BbvCI. Triggering CDN T with the fuel strand L_1_′ displaces the duplex L_1_/T_1_ while generating the duplex L_1_/L_1_′ and the free strand T_1_ that hybridizes with the arms associated with constituent BA′. This results in the dynamic transition of CDN T to CDN Q where the constituent BA′ is upregulated. The component B and component T_1_ in the systems are pre-engineered, however, to include functional tethers f and e which upon the stabilization of constituent BA′ self-assemble into Mg^2+^-ion-dependent DNAzyme cleaving F_1_ (FAM)/Q_1_ (BHQ1)-modified substrate (panel III). The L_1_′ triggered dynamic reconfiguration of CDN T to Q yields the duplex L_1_/L_1_′ that includes a pre-engineered nicking site being cleaved by Nt.BbvCI, resulting in the release of L_1_ that displaces T_1_ from constituent BA′, resulting in the transient recovery of CDN Q to CDN T, while separating the Mg^2+^-ion-dependent DNAzyme, associated with BA′. That is, the triggered dynamic reconfiguration of CDN T to CDN Q leads to the transient catalytic operation of the Mg^2+^-ion-dependent DNAzyme which is followed by the fluorescence of the fragment product of the DNAzyme substrate (Figure 12B). Alternatively, treatment of CDN T with the fuel trigger L_2_′ stimulates the displacement of the duplex L_2_/T_2_ to yield L_2_/L_2_′ and the released strand T_2_ that binds to the “arm” of constituent AA′, leading to the dynamic reconfiguration of CDN T to CDN R, where constituents AA′ and BB′ are upregulated and constituents AB′ and BA′ are downregulated. The component A of constituents AA′ and T_2_ is pre-engineered to include the tethers g and h that correspond to G-quadruplex subunit sequences. Thus, the transition of CDN T to CDN R, in the presence of K^+^-ions and hemin, is accompanied by dynamic formation of the hemin/G-quadruplex DNAzyme, being a part of constituent AA′. The resulting hemin/G-quadruplex DNAzyme catalyzes the oxidation of Amplex Red by H_2_O_2_ to form the fluorescent Resorufin that probes the formation of the hemin/G-quadraplex-functionalized constituent AA′. The fuel-triggered duplex L_2_/L_2_′ inducing the transition of CDN T to CDN R is engineered, however, to include in strand L_2_′ a nicking site that is cleaved by Nt.BbvCI. Cleavage of L_2_′ separates the duplex L_2_′/L_2_, and the released L_2_ displaces T_2_ from constituent T_2_/AA′ leading to the recovery of CDN R to CDN T and to the separation of the hemin/G-quadruplex DNAzyme unit associated with constituent AA′ of CDN R. Thus, the L_2_′-triggered reconfiguration of CDN T to CDN R, in the presence of the duplex L_2_/T_2_ and nickase, leads to the dynamic transient operation of the hemin/G-quadruplex DNAzyme (Figure 12C).

## 3 Conclusions

This Review has addressed an important topic in the rapidly developing area of Systems Chemistry by discussing dynamic catalytic systems driven by nucleic acid–protein (enzyme) conjugates and supramolecular nanostructure composed of nucleic acid scaffolds, coupled to proteins or DNAzymes. These included constitutional dynamic networks (CDNs) that guide biocatalytic cascades and feedback-driven internetwork communicated biocatalytic cascades. In addition, nucleic acid/enzyme reaction modules guiding dynamic out-of-equilibrium, transient biocatalytic cascades were demonstrated. Alternatively, the triggered switchable mechanical dynamic reconfiguration features of DNA nanostructures were conjugated to the enzyme or DNAzyme functionalities to yield switchable mechanically driven catalytic processes. This has been introduced by highlighting the design of interlocked supramolecular DNA catenane structure that reconfigures, in the presence of appropriate triggers, into switchable DNAzyme structures and by tailoring a supramolecular DNA tweezers structure conjugated to enzyme/DNAzyme constituents. The triggered dynamic opening and closing of the tweezers scaffold led to the switchable operation of a biocatalytic cascade. Also, origami tiles provided functional frameworks for positioning functional units for dynamic catalysis. This has been introduced with the photochemically “mechanical” activation of a NAD^+^-cofactor-mediated biocatalytic cascade, the rotational, triggered operation of biocatalytic cascade, and the light-induced reversible mechanical unlocking of a nanohole in the origami raft and the use of the nanohole as a confined environment for the switchable operation of a DNAzyme.

While substantial progress in developing DNA-based networks and supramolecular DNA structures for operating dynamic catalytic transformations was achieved, interesting and challenging goals are ahead of us. The design of DNA-based constitutional dynamic networks or dissipative, transient nucleic acid-based networks guiding catalytic processes of enhanced complexities and functionalities is desirable. For example, the design of networks guiding artificial photosynthetic and photocatalytic transformations or dynamically driven transcription processes inducing network-guided selective translation of proteins can be envisaged. Similarly, enhancing the complexity of dissipative nucleic acid-based reaction modules to yield gated or cascaded transient machinery is anticipated to establish dynamic, transient transcription of mRNAs and their translation to target proteins, thus emulating biological gene regulation and transcription. Indeed, recent studies demonstrated that dynamically modulated transcription and protein translation are achievable goals. Nonetheless, in the present study, the dynamically triggered catalytic networks operate in homogeneous, *in vitro* environments. Their integration in cell-like containments, protocells,^127^ is certainly a future challenge. The advances in developing artificial protocell assemblies, such as liposomes,^128,129^ microdroplets,^130^ dendrosomes,^131^ polymersomes,^132^ and hydrogel microcapsules,^133^ provide exciting opportunities to design artificial cells.

Beyond the significant basic and fundamental issues related to dynamic catalytic networks and structures, the identification of practical applications of these concepts is most important. In fact, substantial research efforts were directed to the development of stimuli-responsive materials and their applications for sensing, imaging, controlled release, and switchable material properties. Integrating the elements of dynamic control over stimuli-responsive functions of materials is anticipated to yield temporally modulated, signal-triggered, materials for diverse analytical (sensing and imaging) and medical applications, such as controlled drug release or sense-and-treat systems. Indeed, the control over three stiffness states of a DNA hydrogel matrix by integrated constitutional dynamic network has been recently exemplified.^80^ Coupling of catalysts to such systems is anticipated to yield dynamic and transient control over material properties.
